# Enhanced transfer of organic matter to higher trophic levels caused by ocean acidification and its implications for export production: A mass balance approach

**DOI:** 10.1371/journal.pone.0197502

**Published:** 2018-05-25

**Authors:** Tim Boxhammer, Jan Taucher, Lennart T. Bach, Eric P. Achterberg, María Algueró-Muñiz, Jessica Bellworthy, Jan Czerny, Mario Esposito, Mathias Haunost, Dana Hellemann, Andrea Ludwig, Jaw C. Yong, Maren Zark, Ulf Riebesell, Leif G. Anderson

**Affiliations:** 1 GEOMAR Helmholtz Centre for Ocean Research Kiel, Kiel, Germany; 2 Alfred Wegener Institute Helmholtz Centre for Polar and Marine Research, Biological Institute Helgoland, Helgoland, Germany; 3 Ocean and Earth Sciences, University of Southampton, Southampton, United Kingdom; 4 Department of Environmental Sciences, University of Helsinki, Helsinki, Finland; 5 Institute for Chemistry and Biology of the Marine Environment (ICBM), Research Group for Marine Geochemistry (ICBM-MPI Bridging Group), Carl von Ossietzky University, Oldenburg, Germany; 6 Department of Marine Sciences, University of Gothenburg, Gothenburg, Sweden; Auckland University of Technology, NEW ZEALAND

## Abstract

Ongoing acidification of the ocean through uptake of anthropogenic CO_2_ is known to affect marine biota and ecosystems with largely unknown consequences for marine food webs. Changes in food web structure have the potential to alter trophic transfer, partitioning, and biogeochemical cycling of elements in the ocean. Here we investigated the impact of realistic end-of-the-century CO_2_ concentrations on the development and partitioning of the carbon, nitrogen, phosphorus, and silica pools in a coastal pelagic ecosystem (Gullmar Fjord, Sweden). We covered the entire winter-to-summer plankton succession (100 days) in two sets of five pelagic mesocosms, with one set being CO_2_ enriched (~760 μatm *p*CO_2_) and the other one left at ambient CO_2_ concentrations. Elemental mass balances were calculated and we highlight important challenges and uncertainties we have faced in the closed mesocosm system. Our key observations under high CO_2_ were: (1) A significantly amplified transfer of carbon, nitrogen, and phosphorus from primary producers to higher trophic levels, during times of regenerated primary production. (2) A prolonged retention of all three elements in the pelagic food web that significantly reduced nitrogen and phosphorus sedimentation by about 11 and 9%, respectively. (3) A positive trend in carbon fixation (relative to nitrogen) that appeared in the particulate matter pool as well as the downward particle flux. This excess carbon counteracted a potential reduction in carbon sedimentation that could have been expected from patterns of nitrogen and phosphorus fluxes. Our findings highlight the potential for ocean acidification to alter partitioning and cycling of carbon and nutrients in the surface ocean but also show that impacts are temporarily variable and likely depending upon the structure of the plankton food web.

## 1. Introduction

The ocean is a major sink for anthropogenic carbon dioxide (CO_2_) by absorbing more than 2 Pg carbon per year from the atmosphere [1,2]. This uptake of atmospheric CO_2_ leads to both carbonation (increasing CO_2_ concentration) and acidification (decreasing seawater pH) of the surface ocean [3,4]. Changes of both environmental factors are expected to impact marine biota from the organism [5] to the ecosystem level [6,7]. Phytoplankton groups belonging to the picoeukaryotes will likely benefit from increased inorganic carbon availability [8,9], while calcifying phyto- and zooplankton groups such as coccolithophores or pteropods will likely be impaired by decreasing seawater pH and changes in seawater carbonate chemistry [10,11]. Presumed shifts in plankton community composition, e.g. to smaller (medium-sized) phytoplankton organisms [12] with different elemental stoichiometry can modify marine element cycling [13–15]. Recent studies have further revealed the potential of CO_2_ to alter the partitioning of carbon between dissolved and particulate organic matter pools in the euphotic ocean zone [16–18]. Increasing proportions of dissolved organic carbon can stimulate bacterial growth and recycling of organic matter [17,19,20], but are also known to promote particle formation and organic matter export by increasing particle stickiness [19,21]. While our knowledge about the impact of CO_2_ on carbon cycling in the ocean is continuously growing, the potential effects on cycling of macronutrients (inorganic nitrogen, phosphorus, and silica) through changes in the marine food webs require more in-depth investigation. In fact, the partitioning of macronutrients between different pools and trophic levels determines their turnover rates and can thereby feedback on ecosystem structure and functioning [22]. For instance changes in stoichiometry and fatty acid composition of primary producers as a consequence of increasing CO_2_ have already been shown to impact mesozooplankton reproduction and development [23,24]. This implies direct consequences for element cycling within the ocean’s food webs.

Calculating the mass balance of carbon and macronutrients is one of the best approaches to estimate their partitioning and cycling. However, such approaches are prone to high uncertainties in open ocean regions. Availability of essential parameters (e.g. gas exchange of CO_2_ or sinking particulate matter) is often limited, while vertical mixing and lateral advection permanently exchange the investigated water masses. Pelagic mesocosms have the advantage of isolating a water mass from the surrounding ocean and hence allow us to investigate natural plankton assemblages of several trophic levels at close to natural conditions. The enclosed water bodies can be characterised with respect to element pools and plankton community, while repetitive sampling of the same water parcel allows monitoring of changes over long timescales and successive phases of plankton development.

Here we present results from a pelagic *in situ* mesocosm CO_2_ perturbation study in Gullmar Fjord (Sweden) covering the full winter-to-summer plankton succession typical for the coastal sea in mid-latitudes. The mid-latitude regions are of particular importance to global element cycling due to the annual formation of large phytoplankton spring blooms characterised by high export efficiency [25]. We monitored the enclosed plankton communities (from viruses to fish larvae) over more than 100 days in two sets of five mesocosms representing ambient and projected year 2100 *p*CO_2_ (partial pressure of CO_2_), respectively [26]. Element pools of carbon, nitrogen, phosphorus, and silica (C, N, P, and Si) were measured to compute mass balances and estimates of net community production, thereby assessing the impact of ocean acidification on the partitioning and cycling of major elements within the ocean surface layer.

## 2. Materials and methods

### 2.1 Mesocosm setup and maintenance

**Fig 1:**
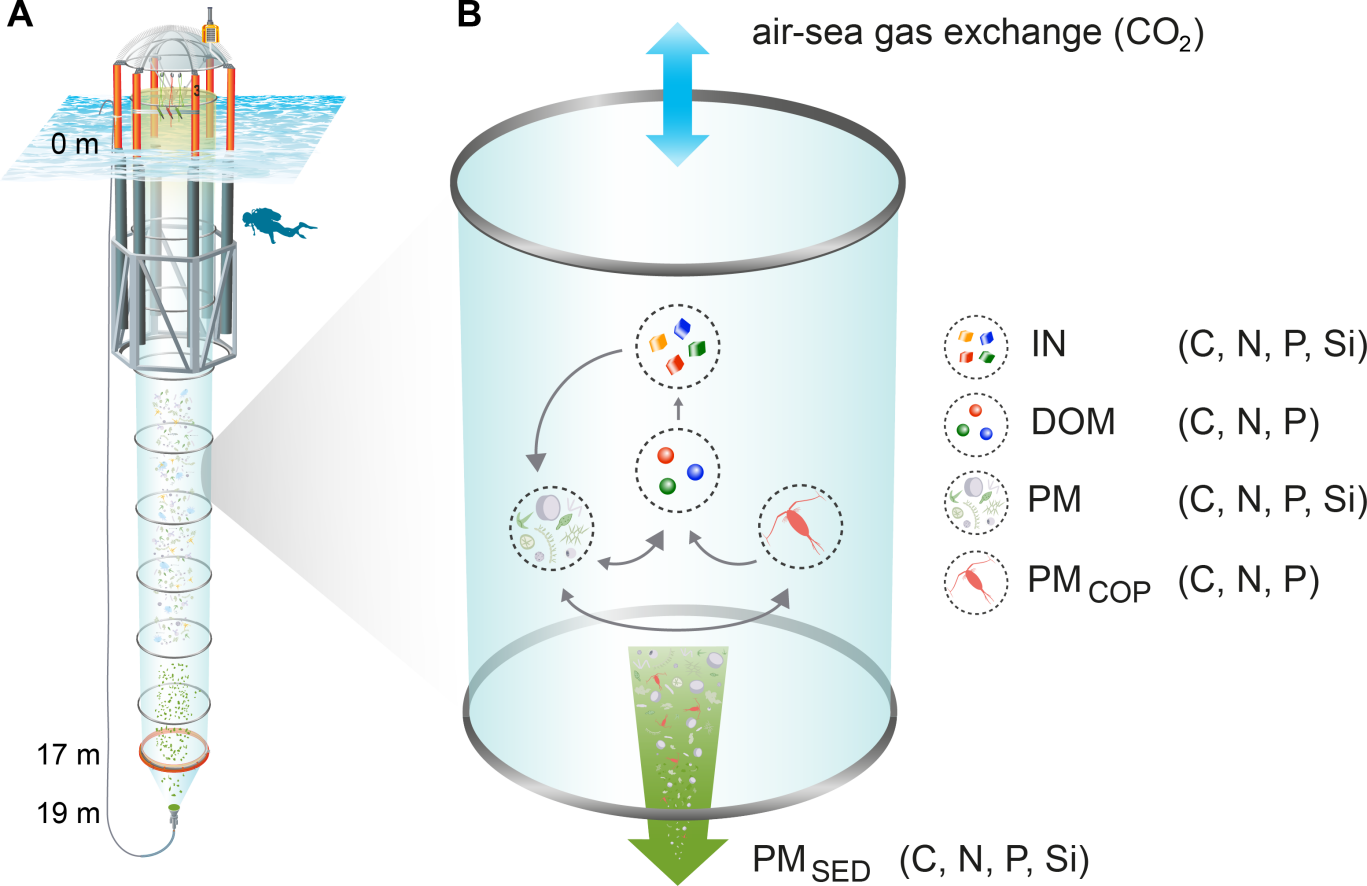
KOSMOS mesocosm unit and conceptual figure of element pools and fluxes. (A) Schematic illustration of a KOSMOS unit, including the floatation frame at the sea surface and the enclosure bag reaching down to the sediment trap at the bottom. (B) Element pools (inorganic nutrients [IN], dissolved organic matter [DOM], particulate matter [PM], particulate matter of copepods [PM_COP_]) and fluxes (air-sea gas exchange of CO_2_, sedimentation of particulate matter [PM_SED_]) included in the mass balance calculations of carbon, nitrogen, phosphorus, and silica (C, N, P, and Si). Grey arrows indicate exchange between the individual element pools in the water column. Illustration of the KOSMOS unit modified from Rita Erven (GEOMAR).

Ten “Kiel Off-Shore Mesocosms for Ocean Simulations” (KOSMOS; [27]) were deployed on January 29, 2013 in Gullmar Fjord on the west coast of Sweden (58.26635°N, 11.47832°E). Permission for the study location was granted by the Längsstyrelsen Västra Götalands Län (reference no: 258-39615-2012) and by the owner of the adjacent private property (Lysekil Skaftö 1:27). Sea ice drift and technical problems described in Bach et al. [26] delayed the start of the experiment until March 7 (day -2 = t_**-2**_, i.e. 2 days before homogenization of the water column; see Sect. 2.2). Each cylindrical mesocosm bag (2 m diameter) enclosed a 17 m deep water column, sealed at the bottom end by a two meter long, funnel-shaped sediment trap (Fig 1A).

**Fig 2:**
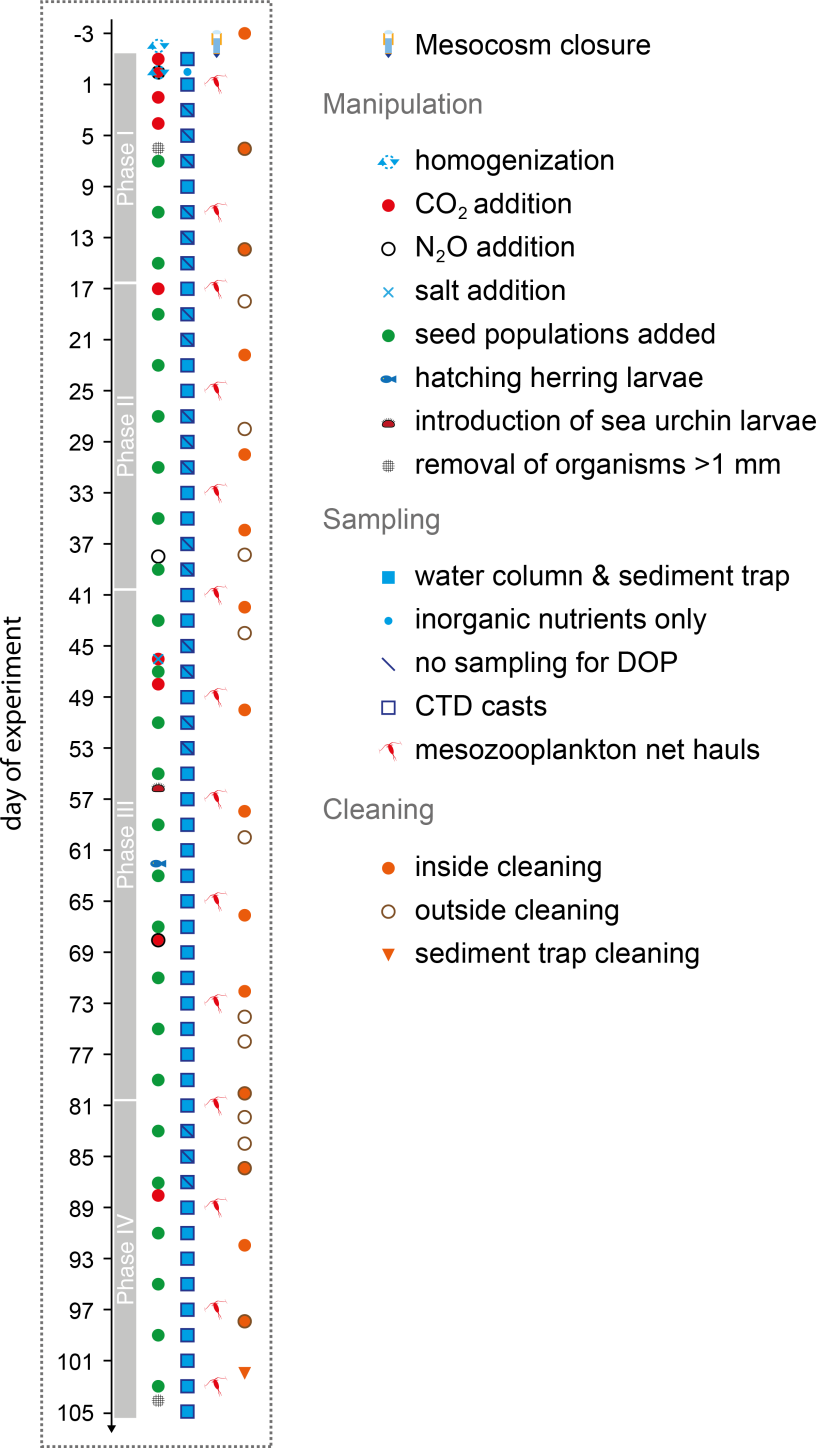
Manipulation, sampling, and maintenance schedule. Days of experiment are relative to the day of water column homogenization (day 0 = t_**0**_).

Enclosed nekton and large mesozooplankton (e.g. fish larvae or jelly fish) were removed during the initial period of the study by a full-diameter-size net (1 mm mesh) that was pulled through each mesocosm (t_**6**_; Fig 2). Samples relevant for mass balancing of elements were taken over a period of more than 100 days until June 22 (t_**105**_; Fig 2). Biofilm formation on the inner and outer walls of the cylindrical mesocosm bags was prevented by regular cleaning [26] (Fig 2). Settled material adhering to the inner surface of the sediment trap funnels was removed at the very end of the experiment (t_**102**_). A detailed description of the study site, the initiation of the experiment, and mesocosm cleaning can be found in Bach et al. [26].

### 2.2 System manipulations and volume determination

The natural salinity gradient inside the mesocosms was removed by injecting air to the bottom of the enclosures in two stages (t_**-2**_ and t_**0**_; Fig 2; [26]). A ‘high CO_2_ treatment’ of initially 961 μatm *p*CO_2_ (t_**5**_) was established in five of the ten mesocosms (M2, M4, M6, M7, M8) by stepwise addition of CO_2_-saturated seawater (t_**-1**_, t_**0**_, t_**2**_, t_**4**_). The other five mesocosms served as untreated controls (M1, M3, M5, M9, M10), representing ambient CO_2_ conditions. *p*CO_2_ levels were re-adjusted four times in the high CO_2_ mesocosms (t_**17**_, t_**46**_ + t_**48**_, t_**68**_, t_**88**_; Fig 2) to counteract the loss from outgassing and biological uptake.

Seed populations of organisms from the surrounding fjord were introduced to the mesocosms by adding 22 L of fjord water on every fourth day (Fig 2). In total, the regular fjord water additions summed up to about 1% of the mesocosms’ volume [26]. In early May, we introduced green sea-urchin larvae (*Strongylocentrotus droebachiensis* [Müller, 1776]; t_**56**_) and herring eggs (*Clupea harengus* [Linnaeus, 1758]; t_**48**_), that hatched two weeks later on t_**62**_. Animal welfare of herring larvae was assured according to guidelines of the ethics permit (no 332–2012). The species *C*. *harengus* is not endangered and specimens were anaesthetised with MS-222 before handling and fixation to reduce stress to a minimum.

The volume of each mesocosm was determined by adding a known amount of calibrated sodium chloride brine solution and by measuring the salinity increase as described in Czerny et al. [28]. The brine solution was evenly dispersed inside the mesocosms on April 24 (t_**46**_), elevating salinity by about 0.1 units from on average 29.2 to 29.3. Mesocosm volumes were converted from kilograms of seawater to litres using individual seawater density of each mesocosm on t_**46**_.

### 2.3 Sampling procedures and CTD operations

The mesocosm water columns and sediment traps were sampled every second day starting at t_**-1**_ with the exception of one additional inorganic nutrient sampling on t_**2**_ (Fig 2). The sediment traps at 19 m water depth were emptied with a vacuum system following Boxhammer et al. [29]. Water column samples were taken with depth-integrating water samplers (IWS, Hydro-Bios) which collected equal amounts of water from all depth levels between 0 and 17 m. Samples sensitive for contamination or gas exchange such as inorganic nutrients (including dissolved inorganic nitrogen (DIN = nitrate (NO_3_^-^) + nitrite (NO_2_^-^) + ammonium (NH_4_^+^)), phosphorus (DIP = phosphate (PO_4_^3-^)) and silica (DSi = Si(OH)_4_)), dissolved organic matter (DOM; DOC (carbon), DON (nitrogen), DOP (phosphorus)) and carbonate chemistry samples (dissolved inorganic carbon (DIC), pH) were directly transferred from the IWS samplers into corresponding sample bottles. DOC/DON samples were gravity filtered through glass fibre filters (pore size 0.7 μm, Whatman) during transfer into pre-combusted glass vials on board of the sampling boats and acidified in the lab (HCl, 25%, analysis grade, Carl Roth) to pH 2 as described in Zark et al. [30]. DOP samples were collected in acid-rinsed polycarbonate bottles (Nalgene) and filtered in the lab trough 0.7 μm (GF/F, Whatman) into low-density polyethylene vials (LDPE, Roth) using gentle vacuum filtration (<200 mbar). Until t_**55**_ DOP samples were only collected on 12 out of 30 sampling days (see Fig 2) and were poisoned with mercury chloride following Kattner [31]. DOP samples collected after t_**55**_ were taken alongside the 48 hours sampling routine (apart from t_**83**_ –t_**87**_) and stored frozen at -20°C.

Carbonate chemistry samples were taken as described in Bach et al. [26] and sterile-filtered (0.2 μm) for a maximum of three days storage (dark and cold) before analysis.

Particulate matter (PM) was sampled from seawater pooled in 10 L carboys that were subsampled within a few hours at *in-situ* water temperatures. Water from these carboys was used for analysis of biogenic silica (BSi), total particulate carbon (TPC), nitrogen (TPN), and phosphorus (TPP), as well as Chlorophyll *a* (Chl*a*) concentrations.

Mesozooplankton was collected with an Apstein net (55 μm mesh size, 17 cm diameter opening) by vertical net hauls (17 to 0 m water depth), representing a sampled volume of about 385 L. We restricted the sampling frequency to every eighth day to minimize the impact on the mesozooplankton community (Fig 2). A subsample of 4% was used for high-resolution plankton imaging with the ZooScan method (see Sect. 2.4.6), while the majority of the sample was preserved with sodium tetraborate-buffered formalin (4% v/v) for taxonomic abundance analyses [32].

CTD casts, providing salinity and temperature profiles, were performed with a CTD60M (Sea & Sun Technology) on every sampling day between 11 a.m. and 3 p.m. (local time; Fig 2), covering a water depth from 0.3 to 18 m.

### 2.4 Sample analysis

#### 2.4.1 Carbonate chemistry measurements and calculations

DIC was determined by colorimetric titration following Johnson et al. [33], with an estimated precision of 3 μmol kg^-1^ (standard deviation of duplicate measurements). Measurement accuracy was ensured by calibration against certified reference materials (CRM, supplied by A. Dickson, Scripps Institution of Oceanography, USA). pH_T_ (total scale) was determined spectrophotometrically, based on the absorption ratio of the sulphonephthalien dye, *m-*cresol purple [34], with a precision of ~0.002 pH units (SD of duplicates), while the accuracy was set by the equilibrium constants of the indicator. *p*CO_2_ was calculated from the combination of pH_T_ and DIC using CO2SYS [35] with the carbonate dissociation constants (K_1_ and K_2_) of Lueker et al. [36]. The input data included salinity, temperature, and inorganic nutrient concentrations (PO_4_^3-^ and Si(OH)_4_).

#### 2.4.2 Inorganic nutrient measurements

Inorganic nutrient samples (NO_3_^-^ + NO_2_^-^, PO_4_^3-^, and Si(OH)_4_,) were filtered as triplicates through 0.45 μm cellulose acetate syringe filters (Whatman) before measuring them with a QuAAtro AutoAnalyzer (Seal Analytical) as described in Bach et al. [26]. When concentrations of NO_3_^-^ + NO_2_^-^ and PO_4_^3-^ dropped below 0.1 μmol L^-1^ (t_**37**_ and t_**35**_, respectively), we switched to using the nanomolar system described by Patey et al. [37]. NH_4_^+^ concentrations were determined according to Holmes et al. [38]. Inorganic nutrient measurements were stopped after t_**95**_ as concentrations were close to or below their detection limits.

#### 2.4.3 DOM measurements

Concentrations of DOC and total dissolved nitrogen (TDN) were analysed of duplicate samples using high-temperature catalytic oxidation on a Shimadzu TOC-VCPH/CPN Total Organic Carbon Analyser, equipped with an ASI-V autosampler and a TNM-1 module for TDN determination as described in Zark et al. [30]. Samples with concentrations of DOC and TDN exceeding the measurement of their duplicate by 30% or more were considered being contaminated and were excluded from the dataset. Measurements from the high and ambient CO_2_ mesocosms were subsequently pooled for identification and removal of outliers using the Dixon-Dean test (p <0.05). DON concentrations were calculated by subtracting the concentration of DIN (see Sect. 2.4.2) from average TDN values.

DOP was converted to orthophosphate by autoclaving for 30 minutes in an oxidizing decomposition solution (Merck, catalogue no. 112936). Concentration of total dissolved phosphate (TDP) was then determined from triplicate subsamples with a QuAAtro AutoAnalyzer (Seal Analytical) as described for PO_4_^3-^ in Sect. 2.4.2. DOP concentrations were calculated by subtracting DIP from TDP concentrations. DON and DOP datasets ended on t_**95**_ because measurements of DIN and DIP were discontinued after this day.

#### 2.4.4 Particulate matter and Chlorophyll *a* measurements

Size fractions of PM smaller and greater than 200 μm (separated with a 200 μm mesh) were collected using gentle vacuum filtration (≤200 mbar) on pre-combusted (6 h at 450°C) glass fibre filters (GF/F, 0.7 μm pore size, Whatman) or cellulose acetate filters (0.65 μm, Whatman) for analysis of TPC, TPN, TPP or BSi, respectively. The thereby collected PM included phytoplankton, small and abundant zooplankton as well as detritus, but essentially no mesozooplankton, such as copepodites or adult copepods (see also Sect. 2.4.6). Glass fibre filters were stored at -20°C in pre-combusted (6 h at 450°C) glass petri dishes until analysis, while cellulose acetate filters were also frozen at -20°C but stored in plastic petri dishes. TPC/TPN filters were oven-dried over night at 60°C, packed in tin foil and analysed alongside blank filters on an acetanilide calibrated CN analyser following Sharp [39]. We refrained from acidifying the filters to remove inorganic C, as pelagic calcifying organisms were very low in abundance. Accordingly, all particulate C data are presented as TPC but are assumed to represent particulate organic carbon (POC). TPP collected on the filters was converted to orthophosphate as described for TDP in Sect. 2.4.3. Concentration of inorganic phosphate was then determined spectrophotometrically according to Hansen and Koroleff [40]. BSi was leached from the collected particulate matter by alkaline pulping with 0.1 M NaOH at 85°C. After 135 minutes the leaching process was terminated with 0.05 M H_2_SO_4_ and DSi was measured by spectrophotometry following Hansen and Koroleff [40]. If not indicated differently, presented PM values are the sum of the two measured size fractions (< and >200 μm). Exceptions are TPP and BSi samples that were filtered as bulk samples before t_**7**_ and t_**29**_, respectively. BSi data of t_**29**_ were removed from the dataset due to a systematic error made during size fractionation on this specific day.

Water column samples for Chl*a* concentration analysis were filtered as described for PM, taking care to minimize light exposure during filtration. Chl*a* content of the collected particles was extracted and analysed by high-performance liquid chromatography (HPLC) as described in Bach et al. [26].

#### 2.4.5 Elemental analysis of sediment trap samples

The sediment trap samples were collected in 5 L Schott Duran glass bottles. To separate PM from bulk seawater, particles were concentrated by flocculation and coagulation with ferric chloride (FeCl_3_) as described by Boxhammer et al. [29]. Briefly, FeCl_3_ and NaOH (for pH stabilisation) were added simultaneously to the well-stirred samples. The clear supernatant water was removed after one hour of particle sedimentation. Mean concentration efficiency of this method was 99.6% with respect to samples’ TPC content [29]. The concentrated samples were centrifuged, deep-frozen at -30°C and lyophilised for 72 hours. The desiccated material was then ground in a ball mill to a homogeneous powder of 2–60 μm particle size [29]. TPC, TPN, TPP, and BSi content of the finely ground sample material was determined from subsamples of 1–2 mg as described for PM of water column samples (see Sect. 2.4.4). The cumulative mass flux of all four elements was expressed in μmol L^-1^ by dividing the summed up mass flux by the calculated mesocosm volumes (Sect. 2.2).

From May 25 (t_**77**_) onwards we screened the freshly taken samples for dead herring larvae that hatched inside the mesocosms on t_**62**_ (see Sect. 2.2). All larvae found were removed for separate analysis, thus they did not contribute to the vertical flux.

#### 2.4.6 Calculation of mesozooplankton biomass

Biomass of the mesozooplankton community was calculated based on abundance data obtained from counting with a stereomicroscope [32]. The community was strongly dominated by the copepod species *Pseudocalanus acuspes* (Giesbrecht, 1881), which represented about 97% of the mesozooplankton counts. Therefore, we only considered copepod biomass for mesozooplankton PM. Copepod nauplii were sufficiently abundant (up to 100 ind. L^-1^) to be sampled quantitatively on PM filters (Sect. 2.4.4). Adult copepods and copepodites, however, were much lower in abundance and naturally escape sampling by the IWS. Thus they were not represented in PM analysis. To avoid double counting of nauplii biomass, only adult copepod and copepodite biomass were included in the calculation of copepod PM (PM_COP_). We applied the image-based ZooScan approach to estimate biomass for the different copepod size classes [41], since biomass measurements of individual organisms have not been conducted. Therefore, subsamples from the mesozooplankton net tows (4% of the total sample) were evenly distributed on a flat-bed scanner (Perfection Pro V750, Epson) to provide high-resolution images (10.6 μm pixel size) of all particles and organisms in the sample. Subsequent image processing with ZooProcess [41] provided a large number of variables for object characterization, including several measures of size such as length or area. For estimation of copepod biomass we then converted measured area of each individual imaged organism to dry-weight (dw) by applying the empirical relationship of [42]:

$$dw=43.97*{area}^{1.52}$$ (1)

The dry-weight was subsequently converted to C and N content (μmol) using the data for body mass composition of zooplankton from Kiørboe [43]. For copepods, the applied C:dw and N:dw ratios were 0.48 and 0.10, respectively. The resulting conversion factors for C and N biomass per individual organism were applied to the complete time series of abundance data for adult copepods and copepodites. P content was calculated using a conversion factor of C:P of 52:1 derived from *Pseudocalanus* sp. caught in Oslofjord (Norway) during the same time of the year (average ratio of individuals caught between March and May) by Gismervik [44].

Similar procedures for image-based biomass estimation of mesozooplankton have been applied in previous studies and showed generally reliable results [45–47]. It should be noted, however, that this approach assumes constant size ranges of copepod life stages and can thus not account for shifts in size structure within a community or population.

### 2.5 Calculation of net changes in element pools and net community production

The relevant pools for mass balancing C, N, P, and Si are dissolved inorganic nutrients (IN_C/N/P/Si_), dissolved organic matter (DOM_C/N/P_), suspended particulate matter (PM_C/N/P/Si_), and the sum of particulate matter collected in the sediment traps (ΣPM_SED (C/N/P/Si)_). Mesozooplankton, strongly dominated by copepods, was treated as a separate PM pool (Sect. 2.4.6), and defined as PM_C/N/P (COP)_. A summary of all pools and fluxes considered in the mass balances are shown in the conceptual Fig 1B. Net changes of the element pools (IN, DOM, PM, and PM_COP_) were calculated as delta (Δ) values relative to conditions at the start of the experiment. We defined the starting conditions as the average value of the first seven sampling days (t_**-1**_ –t_**11**_). Averaging over this relatively long period was necessary to minimize the influence of data variability. This was well justifiable as relative changes of the element pools were small before t_**13**_ (Sect. 3.1). However, some exceptions (listed in the following text) had to be made for distinct element pools. The first two data points of DSi (t_**-1**_ and t_**1**_) were excluded due to a methodological measurement problem. The reference value of ΔDIC in the high CO_2_ treatment is based on a single sampling day t_**5**_, since before DIC was increased by stepwise CO_2_ additions (Sect. 2.2) and afterwards CO_2_ rapidly outgassed to the atmosphere (super-saturation of the water column). DOC and DON data of t_**-1**_ were removed from the datasets as measurements displayed substantial unexplainable variability with strong impact on calculated starting conditions. Reference values for DOP were calculated from three data points (t_**-1**_, t_**1**_ and t_**9**_), as those days were the only days when DOP was sampled during the initial phase of the experiment (Fig 2). The first mesozooplankton sampling on t_**1**_ served as the reference point for net changes in PM_C/N/P (COP)_. The start and end point of the individual reference periods, as well as the calculated reference values of each element pool within the water column are summarized in Table 1.

**Table 1:** Conditions of the element pools during the reference period of the experiment.

		ambient CO_2_			high CO_2_			t-test
		*reference period*		*reference value*	*reference period*		*reference value*	
		start	end	μmol L^-1^ ± SD	start	end	μmol L^-1^ ± SD	p-value
**IN**	DIC	t_**-1**_	t_**11**_	2079.3 ± 3.2	t_**5**_	t_**5**_	2184.3 ± 4.3	**<0.001**
	DIN	t_**-1**_	t_**11**_	7.0 ± 0.1	t_**-1**_	t_**11**_	6.9 ± 0.1	0.380
	DIP	t_**-1**_	t_**11**_	0.76 ± 0.01	t_**-1**_	t_**11**_	0.76 ± 0.01	0.242
	Si	t_**2**_	t_**11**_	9.9 ± 0.3	t_**2**_	t_**11**_	9.8 ± 0.1	0.572
**DOM**	DOC	t_**1**_	t_**11**_	189.0 ± 10.8	t_**1**_	t_**11**_	190.1 ± 5.7	0.840
	DON	t_**1**_	t_**11**_	8.8 ± 0.6	t_**1**_	t_**11**_	8.9 ± 0.4	0.804
	DOP	t_**-1**_	t_**11**_	0.16 ± 0.02	t_**-1**_	t_**11**_	0.14 ± 0.02	0.238
**PM**	TPC	t_**-1**_	t_**11**_	14.4 ± 0.7	t_**-1**_	t_**11**_	14.7 ± 0.8	0.613
	TPN	t_**-1**_	t_**11**_	1.9 ± 0.1	t_**-1**_	t_**11**_	2.0 ± <0.1	0.554
	TPP	t_**-1**_	t_**11**_	0.08 ± 0.01	t_**-1**_	t_**11**_	0.09 ± 0.01	0.665
	BSi	t_**-1**_	t_**11**_	0.4 ± <0.1	t_**-1**_	t_**11**_	0.4 ± <0.1	0.679
**PM**_**COP**_	TPC_COP_	t_**1**_	t_**1**_	7.5 ± 2.5	t_**1**_	t_**1**_	6.8 ± 0.9	0.590
	TPN_COP_	t_**1**_	t_**1**_	1.4 ± 0.5	t_**1**_	t_**1**_	1.2 ± 0.2	0.590
	TPP_COP_	t_**1**_	t_**1**_	0.14 ± 0.05	t_**1**_	t_**1**_	0.13 ± 0.02	0.590

Reference values of both CO_2_ treatments are average values ± standard deviation (SD) of the indicated reference periods for calculation of net changes in the respective element pools (see Table 2 for abbreviations of the element pools). If start and end point of the reference period are identical, the reference period is limited to only one data point. t-tests performed on average values of all ambient and high CO_2_ mesocosms are indicated by p-values (bold values indicate significant difference, p ≤0.05).

**Table 2:** Colour code, line types, and abbreviations of the different element pools and their calculated net community production.

Colour	Line type	Abbreviation	Element pool	Elements	SF
			/ net community production		(days)
dark grey	solid	IN	inorganic nutrients	C, N, P, Si	2
orange	solid	DOM	dissolved organic matter	C, N, P	*2
green	solid	PM	suspended particulate matter	C, N, P, Si	2
brown	solid	PM_**SED**_	sedimented particulate matter	C, N, P, Si	2
light red	solid	PM_**COP**_	calculated copepod organic matter	C, N, P	8
blue	solid	NCP_**C/N/P (COP)**_ ambient CO_2_	net community production of the element at ambient CO_2_ incl. PM_COP_	C, N, P	*8
blue	dashed	NCP_**C/N/P/Si**_ ambient CO_2_	net community production of the element at ambient CO_2_ excl. PM_COP_	C, N, P, Si	*2
dark red	solid	NCP_**C/N/P (COP)**_ high CO_2_	net community production of the element at high CO_2_ incl. PM_COP_	C, N, P	*8
dark red	dashed	NCP_**C/N/P/Si**_ high CO_2_	net community production of the element at high CO_2_ excl. PM_COP_	C, N, P, Si	*2

Sampling frequency (SF) indicates the time resolution of the respective data set.

*Samples for DOP determination were taken irregularly, reducing the time resolution of DOP, NCP_**P**_, and NCP_**P (COP**)_ (see Sect. 2.3).

Net community production (NCP) is most commonly estimated by measuring the biological drawdown of DIC or NO_3_^-^ [48,49]. In the present study, we derived NCP from the actual build-up of biogenic C, N, P, and Si following Hansell and Carlson [48] and Spilling et al. [18]. This total NCP theoretically equals the cumulative drawdown of inorganic nutrients (ΔIN) and is therefore given in moles per litre and not as a rate. We calculated net community production in (1) high temporal resolution lacking mesozooplankton contribution (Eq 2) and (2) in reduced temporal resolution but including mesozooplankton contribution (Eq 3):

$${NCP}_{C/N/P/Si}={\Delta PM}_{C/N/P/Si}+{\Delta DOM}_{C/N/P}+{\Sigma PM}_{SED\;(C/N/P/Si)}$$ (2)

$${NCP}_{C/N/P\;(COP)}={\Delta PM}_{C/N/P}+{\Delta PM}_{C/N/P\;(COP)}+{\Delta DOM}_{C/N/P}+{\Sigma PM}_{SED\;(C/N/P)}$$ (3)

Thus, NCP_C/N/Si_ was calculated for every second day (Table 2), while NCP_P_ followed the irregular sampling of DOP described in Sect. 2.3 and illustrated in Fig 2. NCP_C/N/P (COP)_ was calculated for usually every 8^th^ day (Table 2) following the mesozooplankton sampling regime (Fig 2).

### 2.6 Data analysis and statistics

Data shown in tables and figures represent average treatment values (ambient and high CO_2_) of the five treatment replicates. Datasets of C and Si pools encompassed the entire duration of the experiment until t_**103**_ and t_**105**_, respectively. Datasets of N and P, however, ended on t_**95**_ as this was the last day where DON and DOP data were available (see Sect. 2.4.3).

Two sample t-tests using *R* software [50] were performed for detection of differences in the initial concentrations of element pools between ambient and high CO_2_ mesocosms (average values used for delta calculations; Table 1).

For detection of CO_2_ treatment effects on net changes of element pools and calculated net community production, univariate permutational analysis of variance (PERMANOVA) tests were run in *R* software [50], using Euclidean distances matrices with 99,999 permutations [51,52]. PERMANOVA was chosen, as assumption of homogeneity of variances was not met for all analysed parameters in all experimental phases. CO_2_ effects were evaluated for average values of each experimental phase (see Sect. 3) or in the case of sedimented PM for cumulative values at the end of the four experimental phases.

## 3. Results and discussion

**Fig 3:**
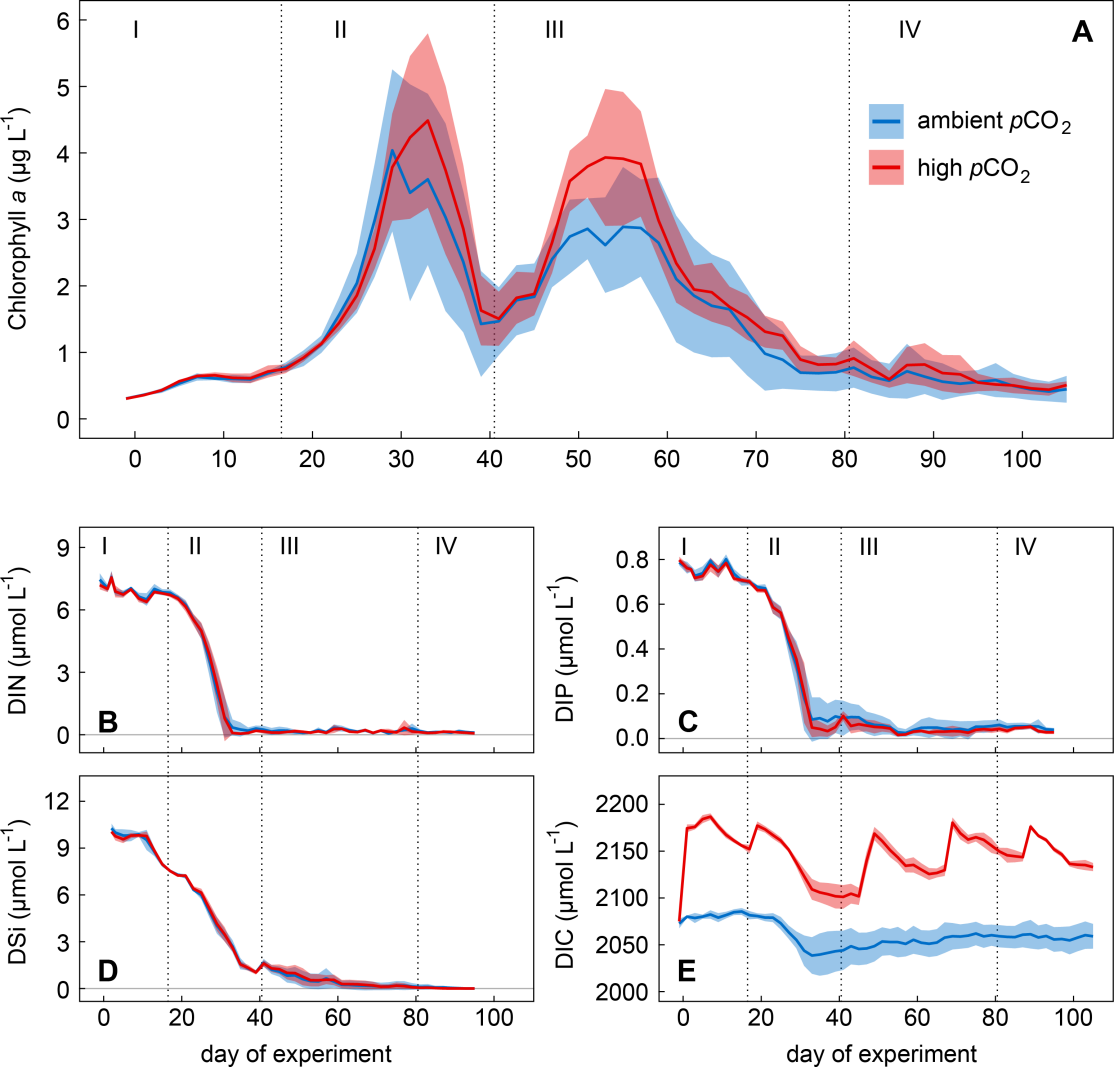
Temporal development of Chlorophyll *a*, inorganic nutrients, and dissolved inorganic carbon. Solid lines show mean values of (A) Chlorophyll *a* (Chl*a*), (B) dissolved inorganic nitrogen (DIN), (C) dissolved inorganic phosphorus (DIP), (D) dissolved silica (DSi), and (E) dissolved inorganic carbon (DIC) in the ambient (blue) and high (red) CO_2_ treatment. Coloured areas indicate the standard deviation of the five treatment replicates. Roman numbers denote the different phases of the experiment.

The experiment was divided into four phases based on the development of Chl*a* concentrations (Fig 3A; see also [26]): Phase I (t_**-1**_ **–**t_**16**_), Phase II (t_**17**_ –t_**40**_), Phase III (t_**41**_ –t_**80**_), Phase IV (t_**81**_ –t_**105**_). These phases were used for the interpretation of net changes in the C, N, P, and Si pools inside the mesocosms (Fig 1B). Average *p*CO_2_ values of the four experimental phases and the entire experiment at ambient and high CO_2_ (t_**-1**_ –t_**105**_) are given in Table 3.

**Table 3:** Overview of the CO_2_ treatments.

Color code	Volume	Phase I		Phase II		Phase III		Phase IV		Phases I–IV	
		t_-1_ –t_16_		t_17_ –t_40_		t_41_ –t_80_		t_81_ –t_105_		t_-1_ –t_105_	
	m^3^ ± SD	*p*CO_2_		*p*CO_2_		*p*CO_2_		*p*CO_2_		*p*CO_2_	
ambient CO_2_	48.2 ± 1.5	366	(–)	329	(–)	367	(–)	447	(+)	377	(–)
high CO_2_	51.0 ± 2.4	762	(+)	641	(+)	747	(+)	878	(+)	756	(+)

Average volume of ambient and high CO_2_ mesocosms (mean ± SD, n = 5) was determined on t_46_ of the experiment. *p*CO_2_ values (μatm) are averages of the four experiment phases (I, II, III, IV) and of the entire experiment (I–IV). The two symbols (+) and (–) represent out- and in-gassing conditions of CO_2_, respectively (presumed atmospheric *p*CO_2_ of 395 μatm).

### 3.1 Temporal development of the C, N, P, and Si pools during the phytoplankton spring-bloom

**Fig 4:**
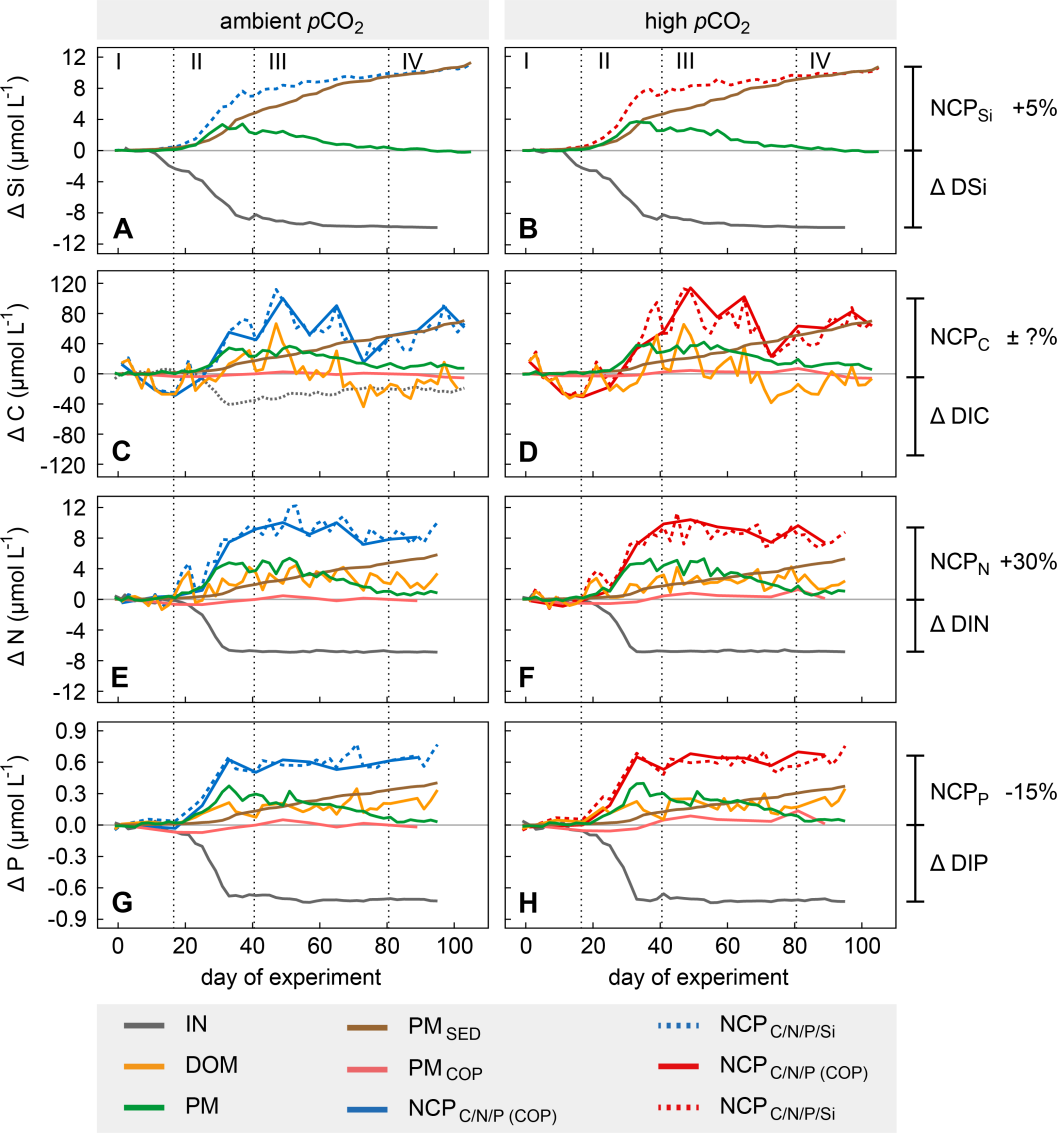
Mass balances of silica, carbon, nitrogen, and phosphorus. Solid and dashed lines indicate temporal net changes (Δ values) of the silica, carbon, nitrogen, and phosphorus (Si, C, N, and P) pools and of their respective net community production as average values of ambient and high CO_2_ mesocosms respectively (see Table 2 for a detailed symbol description). DIC is only included at ambient CO_2_ (grey, dotted line), lacking correction for CO_2_ air-sea gas exchange (see Sect. 3.2). Roman numbers denote the different phases of the experiment. Percentages indicate the approximate discrepancy between net community production and inorganic nutrient consumption during Phases III and IV.

The first (pre-bloom) phase of the experiment was characterised by relatively stable environmental conditions with high concentrations of DIN, DIP, and DSi (~7.0, ~0.76, and ~9.8 μmol L^-1^, respectively; Fig 3B–3D) and short day length [26]. The enclosed water columns were entirely mixed due to thermal convection inside the mesocosm bags [26]. Enclosed plankton assemblages were relatively similar among the ten mesocosms although small differences were detected (see [26]). No significant differences in initial concentrations of inorganic nutrients as well as the other element pools (PM, PM_COP_, DOM) were found between CO_2_ treatments apart from DIC, as a direct consequence of the CO_2_ manipulation (see reference values in Table 1). Net changes in the pools of all four elements (C, N, P, Si) were relatively small underlining the pre-bloom character of Phase I (Fig 4). The decline of DOC in this early phase was not reflected in changes of any other C pool and is therefore more likely associated with sampling induced artefacts than with real changes in the DOC pool. Thus, we do not draw any conclusion from this trend.

The second phase covers the first major build-up and decrease of Chl*a* during the phytoplankton spring-bloom (Fig 3A). Primary production was fuelled by inorganic nutrients that rapidly decreased during the bloom development (Fig 3B–3D). Small silicifiers (2–5 μm, mostly diatoms) as well as the large diatom *Coscinodiscus concinnus* (Smith, 1856; >200 μm) dominated the bloom-forming autotrophic community during this phase (see Bach et al. [53] for detailed information). Low DIN and DIP concentrations limited primary production after t_**31**_ and thus terminated the exponential growth of phytoplankton (Fig 3B and 3C). The decrease of DSi, however, just slowed down according to uptake kinetics of diatoms [54,55]. Thus, DSi was still available in low concentrations after the first bloom (>1 μmol L^-1^ at the end of Phase II; Fig 3D). Peak values of PM were reached between t_**31**_ and t_**37**_ with average net build-up of TPC, TPN, TPP, and BSi of ~33.6, ~4.7, ~0.33 and ~3.3 μmol L^-1^, respectively (Fig 4). Sedimentation of PM of all four elements and build-up of DOM_C/N/P_ started to increase right from the onset of the first phytoplankton bloom (Fig 4). Highest sedimentation rates were observed during the bloom peak, implying a close temporal coupling between primary production and sinking particle flux. In contrast to particulate C, N, and P, the amount of BSi removed from the water column during this period equalled the net build-up in the water column (Fig 4). BSi:C ratios in the sediment trap samples were four times higher than those in the water column (S1 Fig), suggesting a strong decoupling of the two elements when it comes to sinking from the productive surface layer; a phenomenon also observed in the open ocean [56,57].

The strong decline of Chl*a* concentrations at the end of Phase II was much less pronounced in the PM pools (compare Figs 3A and 4). TPC, TPN, and TPP remained relatively high even though Chl*a* strongly decreased. This suggests a highly efficient transfer of autotrophic into heterotrophic biomass and/or non-sinking phytodetritus accumulating in the water column. Indeed, bacterial as well as micro- and mesozooplankton abundances increased parallel to the Chl*a* decrease [32,58].

Phase III encompassed the second and slightly less pronounced build-up and decrease of Chl*a* during the spring-bloom (Fig 3A). Small diatoms and flagellates (2–5 μm), but mainly the giant diatom *C*. *concinnus* (up to 50% Chl*a* contribution) dominated the phytoplankton community during this phase (see [53]). The shift in dominance from small diatom species (<200 μm) to the large cells of *C*. *concinnus* (>200 μm) is clearly reflected in the temporal development of the two size fractions of BSi (S2 Fig). Regenerated N and P, as well as the remaining DSi likely fueled primary production during this second bloom. Peak values of PM were reached between t_**49**_ and t_**53**_ with average net build-up of TPC, TPN, and TPP of ~36.8, ~5.1, and ~0.25 μmol L^-1^, respectively (Fig 4C–4H). A peak in net build-up of BSi was absent due to the high loss through sedimentation and only very low DSi concentrations available (Figs 4A, 4B and 3D). The high variability in DON and DOP concentrations likely masked consumption of both pools by the plankton community (Fig 4E–4H). We suspect that considerable proportions of DON and DOP were rather refractory and only a small fraction of these pools, composed of labile compounds, was used and turned over by bacteria and phytoplankton on time scales that could not be resolved by our 48 h sampling regime. This assumption is consistent with field observations [59,60] and is supported by relatively high background concentrations of (likely refractory) DON and DOP right after mesocosm closure (see Table 1). Labile DOP is known to be recycled within hours to days [59,61], therefore often fuelling primary production under DIP depletion. In contrast to the relatively stable concentrations of DON and DOP (Phases III and IV), DOC concentrations showed a decreasing trend during the second phytoplankton bloom, reaching values lower than the initial ones by the end of Phase III.

Adult copepod and copepodite biomass (PM_C/N/P (COP)_) decreased directly after mesocosm closure (Fig 4C–4H), but increased again during the phytoplankton blooms with highest values reached during and after the second bloom peak in Phase III. Predation by herring larvae that hatched inside the mesocosms (t_**62;**_ see Sect. 2.2) and started feeding on larger mesozooplankton around t_**80**_ were most likely responsible for the decline of PM_C/N/P (COP)_ in the post-bloom Phase IV. The relative change in the PM_C/N/P (COP)_ pool was most pronounced in the P mass balance due to the relatively high P content of copepods. In contrast to primary producers, the PM pool of mesozooplankton is likely not properly accounted for in most mass balance approaches due to the relatively small number of organisms in the sample volumes that are used for PM analysis. We observed a temporal contribution of up to 20% to TPP build-up, which emphasizes that this pool should not be neglected.

The fourth phase (post-bloom) was characterized by typical summer conditions in the coastal mid-latitudes. Inorganic nutrient concentrations were depleted (Fig 3B–3D), the water column was stratified (see [26]), and PM concentrations had almost declined to those of the pre-bloom phase (Fig 4).

### 3.2 Mass balances of Si, C, N, and P

The NCP of all four investigated elements (see Eqs 2 and 3) should in theory match the consumption of their inorganic nutrients over time. This worked out well for Si, where NCP was only slightly overestimated with on average ~5% during Phases III and IV (2^nd^ bloom and post-bloom; Fig 4A and 4B). This is well within the range one would expect from combining measurement uncertainties of three different pools (DSi, BSi, BSi_SED_; Fig 1B). Interestingly, we observed a temporal mismatch of DSi consumption and NCP_Si_ shortly before and during the onset of the spring-bloom (between Phases I and II). Wall growth, a common artefact in enclosure experiments [62–64], can be excluded as a sink for DSi, as mesocosm inside walls were frequently cleaned (see Sect. 2.1; Fig 2) and we have not observed a comparable pattern in the mass balances of N or P (Fig 4E–4H). Thus, we assume that this mismatch at the end of Phase I might be explained by internal storage of DSi in diatom cells, which can contribute up to 50% of their total silica content (see review by Martin-Jézéquel et al. [65]). We observed that one of the dominating diatom taxa during that time, *Arcocellulus* sp. (see [53]), had very fragile frustules that potentially broke during the filtration process for BSi analysis and released internally stored DSi. Apart from this specific period the Si mass balance was virtually closed.

When attempting to calculate the mass balance of C, we faced two major difficulties. These were (1) the unexplainable day-to-day variability in DOC data (up to ~50 μmol L^-1^ within 48 hours; Fig 4C and 4D) and (2) the poorly constrained gas exchange of CO_2_ with the atmosphere. Both made it ultimately impossible to calculate a reasonable mass balance of C. Achieving accurate DOC data in an experimental setup like pelagic mesocosms has shown to be challenging [64], but not impossible [9,66]. Measurement precision and accuracy in the present study was high [30], so that the variability is more likely to originate from artifacts which were induced during sampling. We refrained from smoothing the data by calculating moving averages since potential contaminations can only increase not decrease the mean and would have led to an overestimation of DOC build-up and NCP_C_ (see S3 Fig). However, it should be noted that the strong variability in ΔDOC had a substantial impact on calculated NCP_C_ (Fig 4C and 4D).

To correct DIC for the air-sea flux of CO_2_ we have followed the approach described by Czerny et al. [67], using the injected tracer gas N_2_O to infer the exchange rate (“gas transfer velocity”) of CO_2_. This technique has been shown to yield good estimates of CO_2_ transfer velocity in past mesocosm experiments under relatively stable physical conditions [18,64]. However, in the present study, the hydrographic situation within the mesocosms was highly dynamic with initial thermal circulation of the entire water columns until t_**37**_, followed by variable thermal stratification and surface layer mixing depth (see [26]). The thermocline physically isolated the bottom layer of the mesocosms from the atmosphere and led to pulsed inputs of N_2_O into the surface layer every time the mixing depth changed. This in turn resulted in discontinuous outgassing of N_2_O, which impeded reasonable estimates of N_2_O and consequently CO_2_ gas transfer velocities. Hence, DIC concentrations could not be corrected appropriately for CO_2_ air-sea gas exchange. To illustrate the discrepancy of un-corrected DIC data with NCP_C_ we have included the measured net change in DIC into Fig 4C (dotted grey line, ambient treatment). In Phase IV the cumulative sedimentation of C alone exceeds net drawdown of DIC by a factor of three. Including CO_2_ gas exchange with the atmosphere is therefore clearly crucial for mass balance calculations of C or when net organic C build-up is calculated from DIC drawdown. Hence, the exclusion of the CO_2_ air-sea gas exchange in DIC drawdown [68] should be seen as very critical.

Balancing the NCP of N and P with DIN and DIP drawdown was not as easy as for Si but not as difficult as for C. The offset between inorganic nutrient consumption and NCP during build-up of the first bloom (Phase II) was highly variable in the case of N (-40 to +13%) and relatively constant for P (approx. -7%; Fig 4E–4H). The offset stabilised during Phases III and IV at values of about +30% and -15% for N and P, respectively, when the phytoplankton community had taken up all inorganic nutrients. NCP_N_ can theoretically be increased above DIN consumption by N_2_-fixation, a significant external N source in the close-by Baltic Sea [69–71]. However, this explanation for the overestimated NCP_N_ was excluded in the present study, as the corresponding organisms (diazotrophic cyanobacteria) were not present in the mesocosms. The pronounced overestimation of NCP_N_ is therefore more likely a result of accumulated measurement inaccuracies of the N pools. Similar to DOC, build-up of DON showed strong variability of up to 4 μmol L^-1^ within 48 hours, not reflected in any other N pool (Fig 4E and 4F). Thus, the DON pool was the source of the largest uncertainty within the N mass balance.

The underestimation of NCP_P_ was unexpected as uncertainties in sampling of DOM and PM (e.g. clogging of filters or bursting phytoplankton cells) rather result in a certain overestimation of the two pools, than leading to their underestimation (Fig 4G and 4H). The discrepancy in DIP consumption and NCP_P_ might be caused by variability in DIP measurements during the reference period used for calculation of net changes (see Table 1 and Sect. 2.5). During this period, DIP concentrations varied by about 0.1 μmol L^-1^ within 48 hours with only low primary production going on [72]. A potential overestimation of the background concentration of DIP by about 0.1 μmol L^-1^ could have led to an overestimated consumption of DIP, possibly explaining the observed offset in the P mass balance.

Altogether, our study has shown that mass balance calculations of elements in marine ecosystems are challenging even in enclosed mesocosm systems with discrete measurements of all relevant parameters. Precise determination of the DOM pools and in the case of C the accurate correction of DIC by the CO_2_ air-sea gas exchange turned out to be most critical. This highlights the enormous challenge of mass balancing elements in open systems (e.g. the coastal ocean, estuaries or eddies) where even more uncertainties emerge due to permanent exchange of water masses.

### 3.3 Impact of CO_2_ on partitioning of C, N, and P

**Fig 5:**
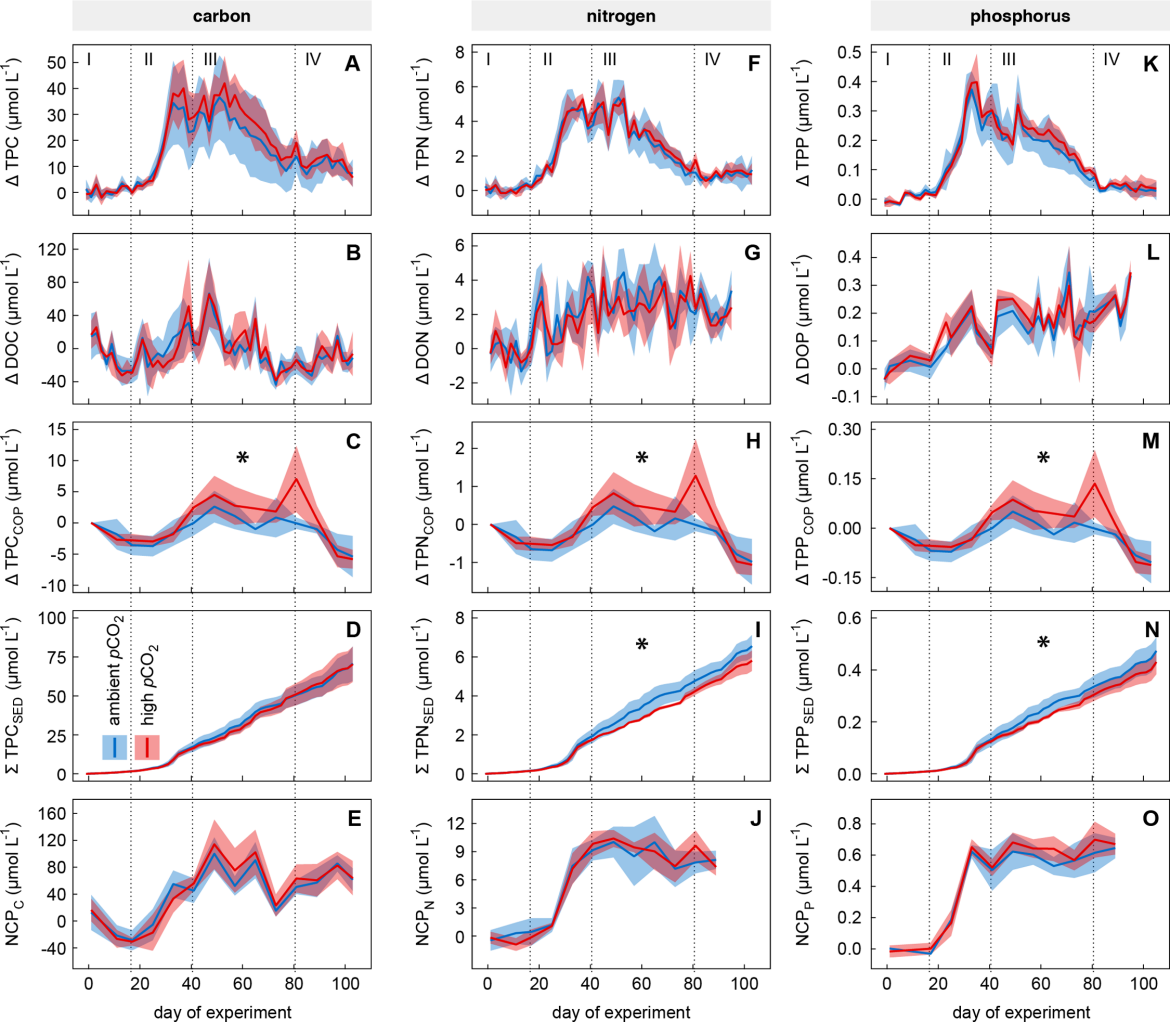
Time course of net changes of the element pools at ambient and high CO_2_. Solid lines indicate temporal net changes (Δ values) of the element pools and net community production of (A–E) carbon, (F–J) nitrogen, and (K–O) phosphorus as average values of the ambient (blue) and high (red) CO_2_ mesocosms. Coloured areas indicate the standard deviation of replicated (n = 5) treatments. Roman numbers denote the four different phases of the experiment. Black asterisks identify significant CO_2_ effects (PERMANOVA, p <0.05).

**Fig 6:**
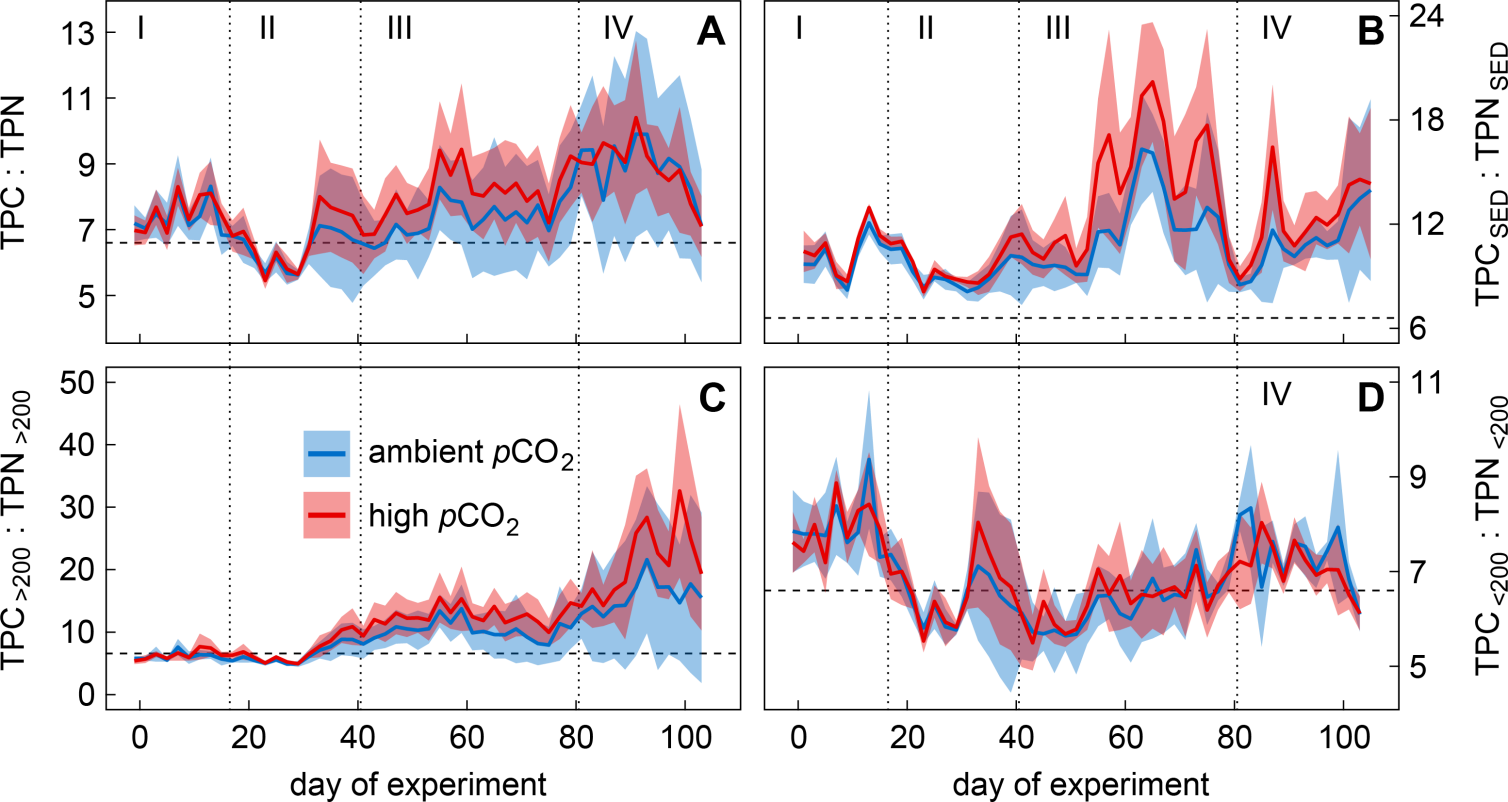
Time course the particulate carbon to nitrogen ratio at ambient and high CO_2_. Solid lines show mean values of the particulate carbon (TPC) to nitrogen (TPN) ratio in (A) the water column, (B) collected sediment trap samples, and of the suspended particle size fractions (C) larger and (D) smaller than 200 μm in the ambient (blue) and high (red) CO_2_ treatment. Coloured areas indicate standard deviation of replicated (n = 5) treatments. Roman numbers denote the four different phases of the experiment. Vertical dashed lines represent the Redfield ratio of carbon to nitrogen (6.6).

TPC was the only PM pool influenced by increased CO_2_ (Fig 5A, 5F and 5K). We observed a positive trend in TPC build-up at high CO_2_ during both phytoplankton blooms (Phases II and III; up to 7 and 9 μmol L^-1^, respectively), although this observation was statistically non-significant due to high within-treatment variability (Fig 5A, Table 4). The tendency of increased C-fixation was likely caused by enhanced ‘carbon overconsumption’ [73,74], which was also indicated by a positive trend in the C:N ratio of particulate matter at high CO_2_ during both phytoplankton blooms (Fig 6A; non-significant in all Phases, see Table 5). The observed trend in the C:N ratio was most prominent in the particle size fraction larger than 200 μm, which was mainly constituted by the large diatom *C*. *concinnus* (Fig 6C). Thus, the dominance of *C*. *concinnus* during the second phytoplankton bloom (see Sect. 3.1 and [53]) explains the influence of the particle size fraction larger 200 μm on the bulk PM C:N ratio during this time (Phase III). The CO_2_-dependent C:N signal in the water column was also found in sinking PM (Fig 6B), indicating that the excess C fixed by *C*. *concinnus* (size fraction >200 μm) was not transferred into higher trophic levels or re-mineralized by bacteria in the water column. Instead this C was removed from the water column by sinking *C*. *concinnus* cells.

**Table 4:** Tested CO_2_ effects on selected pools and net community production.

Parameter	ambient CO_2_	high CO_2_	SS	Pseudo-F	p (perm)
	μmol L^-1^ ± SD	μmol L^-1^ ± SD			
**ΔTPC**					
I	0.6 ± 0.4	0.5 ± 0.3	0.001	0.010	0.897
II	16.4 ± 7.1	17.6 ± 4.6	3.536	0.100	0.755
III	22.9 ± 12.4	27.6 ± 7.1	55.450	0.540	0.477
IV	10.3 ± 5.0	11.8 ± 4.3	6.015	0.280	0.587
**ΔTPC**_COP_					
I	-0.9 ± 1.2	-1.4 ± 0.5	0.478	0.561	0.595
II	-3.0 ± 1.7	-2.5 ± 0.9	0.496	0.277	0.619
III	0.7 ± 1.0	2.8 ± 1.6	10.588	5.946	^**(+)**^ **0.047**
t_**35 –t89**_	0.4 ± 0.8	3.1 ± 1.8	18.399	9.145	^**(+)**^ **0.016**
IV	-2.7 ± 1.7	-0.9 ± 1.3	8.187	3.507	0.087
**ΔTPN**_COP_					
I	-0.2 ± 0.2	-0.3 ± 0.1	0.016	0.561	0.595
II	-0.5 ± 0.3	-0.5 ± 0.2	0.016	0.277	0.621
III	0.1 ± 0.2	0.5 ± 0.3	0.351	5.946	^**(+)**^ **0.047**
t_**35 –t89**_	0.1 ± 0.1	0.6 ± 0.3	0.589	9.014	^**(+)**^ **0.016**
IV	-0.5 ± 0.3	-0.2 ± 0.2	0.272	3.507	0.088
**ΔTPP**_COP_					
I	-0.02 ± 0.02	-0.03 ± 0.01	<0.001	0.561	0.595
II	-0.06 ± 0.03	-0.05 ± 0.02	<0.001	0.277	0.618
III	0.01 ± 0.02	0.05 ± 0.03	0.004	5.946	^**(+)**^ **0.046**
t_**35 –t89**_	0.01 ± 0.02	0.06 ± 0.04	0.007	9.145	^**(+)**^ **0.015**
IV	-0.05 ± 0.03	-0.02 ± 0.03	0.003	3.507	0.088
**ΣTPN**_SED_					
t_**15**_	0.1 ± <0.1	0.1 ± <0.1	<0.001	0.939	0.358
t_**39**_	1.8 ± 0.3	1.7 ± 0.1	0.065	1.074	0.355
t_**69**_	4.1 ± 0.5	3.4 ± 0.1	1.116	8.352	^**(–)**^ **0.047**
t_**79**_	4.6 ± 0.5	4.1 ± 0.2	0.725	4.883	0.063
t_**105**_	7.0 ± 0.6	6.3 ± 0.6	1.438	4.367	0.088
**ΣTPP**_SED_					
t_**15**_	0.01 ± <0.01	0.01 ± <0.01	<0.001	0.083	0.786
t_**39**_	0.12 ± 0.03	0.12 ± 0.01	<0.001	0.196	0.683
t_**69**_	0.29 ± 0.03	0.25 ± 0.01	0.004	7.903	^**(–)**^ **0.040**
t_**79**_	0.33 ± 0.04	0.30 ± 0.02	0.002	2.297	0.174
t_**105**_	0.47 ± 0.05	0.43 ± 0.05	0.005	1.844	0.189
**NCP**_C_					
I	-4.5 ± 14.4	-5.1 ± 13.6	1.040	0.005	0.939
II	7.0 ± 16.0	-4.9 ± 12.3	352.050	1.741	0.239
III	60.6 ± 9.3	74.1 ± 17.6	456.040	2.305	0.152
IV	63.7 ± 6.8	67.6 ± 15.9	36.600	0.245	0.653

Values are average values of the different phases (I–IV) in the ambient and high CO_2_ treatments ± standard deviation (SD). Effects of CO_2_ were assessed by PERMANOVA, giving the sum of squares (SS), the F value by permutation (Pseudo-F), and the p-value (p (perm)). Significant effects detected are highlighted in bold, while positive or negative trends are indicated by ^(+)^ and ^(-)^, respectively.

**Table 5:** Tested CO_2_ effects on the total particulate carbon to nitrogen ratio.

Parameter	ambient CO_2_	high CO_2_	SS	Pseudo-F	p (perm)
	mol:mol ± SD	mol:mol ± SD			
**C:N _BULK_**					
P I	7.4 ± 0.2	7.5 ± 0.3	0.027	0.433	0.515
P II	6.4 ± 0.5	6.7 ± 0.4	0.198	0.909	0.353
P III	7.3 ± 1.3	8.1 ± 1.1	1.438	1.042	0.324
P IV	8.9 ± 2.3	8.9 ± 1.2	0.001	<0.001	0.984
**C:N _<200 μm_**					
P I	8.0 ± 0.3	7.9 ± 0.3	0.002	0.029	0.864
P II	6.5 ± 0.5	6.6 ± 0.4	0.055	0.250	0.614
P III	6.3 ± 0.5	6.4 ± 0.4	0.029	0.132	0.634
P IV	7.4 ± 0.5	7.1 ± 0.2	0.155	1.241	0.315
**C:N _>200 μm_**					
P I	6.2 ± 0.3	6.4 ± 0.4	0.110	1.035	0.332
P II	6.3 ± 0.7	7.0 ± 0.6	1.242	2.685	0.158
P III	10.2 ± 3.0	12.5 ± 2.7	12.757	1.583	0.302
P IV	15.8 ± 9.6	21.2 ± 5.8	74.100	1.183	0.308
**C:N _SED_**					
P I	10.1 ± 0.5	10.6 ± 0.4	0.661	3.409	0.105
P II	9.2 ± 0.4	9.6 ± 0.4	0.399	2.683	0.143
P III	11.6 ± 1.6	13.9 ± 2.5	14.315	3.139	0.135
P IV	10.8 ± 1.1	12.2 ± 1.1	4.699	3.759	0.095

Values are average values of the different phases (I–IV) in the ambient and high CO_2_ treatments ± standard deviation (SD). Effects of CO_2_ were assessed by PERMANOVA, giving the sum of squares (SS), the F value by permutation (Pseudo-F), and the p-value (p (perm)). Significant effects detected are highlighted in bold, while positive or negative trends are indicated by ^(+)^ and ^(-)^, respectively.

In contrast to other plankton community CO_2_ perturbation studies [16,17], we have not detected an increase in DOC build-up at high CO_2_, although the high variability in the present data set may have masked small differences (Fig 5B).

Surprisingly, we found that copepod biomass was significantly elevated under high CO_2_ during times of regenerated production (Phase III; Table 4). Between the peak of the first phytoplankton bloom and mid of the post-bloom Phase IV (t_**35**_ –t_**89**_), PM_C/N/P (COP)_ was increased by on average 2.7, 0.5 and 0.05 μmol L^-1^ with respect to TPC_COP_, TPN_COP_, and TPP_COP_ (Fig 5C, 5H and 5M). Enhanced primary production at high CO_2_ that was revealed by Eberlein et al. [72] during the same study must have caused this amplified transfer of biomass from primary producers (phytoplankton) to the higher trophic level of mesozooplankton (see [75]). Increased abundance of zooplankton organisms without a corresponding increase in primary producers and their respective biomass has also been observed in other studies [76]. The disappearance of the CO_2_ effect on copepod biomass (C, N, P) towards the end of the study (Phase IV) can likely be explained by a potential further transfer into biomass of herring larvae. This assumption is supported by (1) the fact that the decrease in copepod biomass in the water column (Fig 5C, 5H and 5M; Phase IV) was not reflected in the downward flux of C, N, and P (Fig 5D, 5I and 5N) and (2) by the fact that the difference in copepod biomass build-up between the two CO_2_ treatments was reflected in an increased survival rate of herring larvae at high CO_2_ by the end of the study (see [77]). The amplified transfer of C, N, and P to higher trophic levels (copepods and likely fish larvae) at high CO_2_ in turn has caused a prolonged retention of biomass in the water column, which significantly reduced the downward flux of N and P (Table 4). Cumulative sedimentation of both elements started to differ between treated and control mesocosms at the same time when CO_2_ driven trends in TPN_COP_ and TPP_COP_ occurred (t_**35**_ onwards; Fig 5H, 5I, 5M and 5N). The difference in cumulative sedimentation of N and P between treatments (Fig 5I and 5N) constantly increased until t_**69**_ (0.7 and 0.04 μmol L^-1^ for N and P, respectively) and remained at this level until the end of the experiment. On t_**105**_ the deposition of N and P at high CO_2_ was reduced by on average ~11 and ~9%, respectively. Due to increasing within-treatment variability during the second half of the study, cumulative sedimentation of both elements was significantly different on t_**69**_ but not on the last day of experiment (t_**105**_; Table 4).

Interestingly, the prolonged retention of biomass within the water column did not affect the downward flux of C (Fig 5D). We assume that the positively influenced relative C content (i.e. C:N ratio) of sinking PM under high CO_2_ (Fig 6B) must have compensated for a theoretically reduced sedimentation of the element. The CO_2_ related trend in the C:N ratio of sinking particles was driven by large amounts of sinking *C*. *concinnus* cells, dominating the downward flux of PM during Phases III and partly IV (see S4 Fig). Hand picked cells from unprocessed sediment trap samples (t_**65**_) were found to have C:N ratios of up to 30:1 in the high CO_2_ mesocosms. The large cell size of *C*. *concinnus* (>200 μm) prevented grazing by the dominating mesozooplankton species *P*. *acuspes*, which excluded transition of its biomass into higher trophic levels and allowed the diatom cells to sink out of the water column.

Our findings show that increased retention of N and P within the pelagic food web under high CO_2_ can lead to a significant and equivalent reduction of their sedimentation. Furthermore, the plankton community composition in the present study has shown that a predator-prey size mismatch between phyto- and mesozooplankton taxa (here *C*. *concinnus* and *P*. *acuspes*) can strongly influence element cycling. Together with changes of phytoplankton C:N ratios, the observed impacts of ocean acidification on element partitioning have the potential to alter cycling of carbon and nutrients in the marine realm.

## 4. Conclusions

In this study we investigated the influence of simulated ocean acidification on the development and partitioning of the C, N, P, and Si pools in a coastal pelagic ecosystem. Our mass balance approach over 100 days, covering a natural winter-to-summer plankton succession, has highlighted important challenges and uncertainties in elemental mass balance calculations, but also revealed significant changes of element pool partitioning under realistic end-of-the-century CO_2_ concentrations (~760 μatm *p*CO_2_):

- Even in a closed mesocosm system we experienced high uncertainties and methodological challenges for our mass balance approach that highlight potential uncertainties in balance calculations of major biogeochemical elements in the open ocean. Accurate determination of the DOM pools and the CO_2_ air-sea gas exchange were most critical in the current study.
- Transfer of C, N, and P from primary producers to higher trophic levels during times of regenerated production was significantly amplified at high CO_2_, leading to prolonged retention of biomass in the water column. Retention of N and P within the pelagic food web resulted in reduced sedimentation of both elements by about 11 and 9%, respectively.
- C-fixation relative to N showed a positive trend at high CO_2_, correlating with the time of inorganic nutrient depletion and the bloom of the large diatom *C*. *concinnus*. The excess C fixed by *C*. *concinnus* was not available for higher trophic levels due to its large cell size (>200 μm) and was removed from the water column by sinking of the diatom cells. This excess C counteracted a potential reduction in C sedimentation that could have been expected from patterns of N and P fluxes.

Even though the observed impacts were temporarily variable and likely dependant on the food web structure, our findings show that ocean acidification has the potential to change the biogeochemical cycles of C, N, and P by retaining C and nutrients in the sea surface food web.

## Supporting information

S1 Fig Time course of the biogenic silica to total particulate carbon ratio. Solid lines show mean values of the biogenic silica (BSi) to particulate carbon (TPC) ratio in (A) the water column and (B) sediment trap samples of the ambient (blue) and high (red) CO_2_ treatment. Coloured areas indicate standard deviation of the replicated (n = 5) treatments. Roman numbers denote the different phases of the experiment.
(PDF)

S2 Fig Time course of different size classes of biogenic silica. Solid lines, dotted lines, and dashed lines represent the three size classes of total biogenic silica (BSi), the fraction >200 μm, and the fraction <200 μm respectively. All lines represent mean values of the (A) ambient and (B) high CO_2_ treatment. Roman numbers denote the different phases of the experiment.
(PDF)

S3 Fig Moving average of dissolved organic carbon and net community production. Dashed lines show net changes of dissolved organic carbon (DOC, yellow) and net community production of carbon (NCP, blue/red) as average values of (A) ambient and (B) high CO_2_ mesocosms. Solid lines of the same colour code show strongly smoothed data (moving average of nine), with an adjusted reference period for calculation of net changes to t_**1**_ –t_**17**_. Accordingly, smoothed data sets do not start before day 9. Roman numbers denote the different phases of the experiment.
(PDF)

S4 Fig High-resolution image of a typical sediment trap sample during Phase III. Section of a high-resolution image, taken from a sediment trap subsample of Mesocosm 4 (high CO_2_) on t_**65**_ (Phase III). The highly abundant round objects are cells of the large diatom *Coscinodiscus concinnus* (Smith, 1856).
(PDF)

## References

[1] Sabine CL, Feely RA, Gruber N, Key RM, Lee K, Bullister JL, et al The oceanic sink for anthropogenic CO2. Science. 2004;305: 367–371. doi: 10.1126/science.1097403 15256665 10.1126/science.1097403

[2] Le Quéré C, Andrew RM, Canadell JG, Sitch S, Korsbakken JI, Peters GP, et al Global carbon budget 2016. Earth Syst Sci Data. 2016;8: 605–649. doi: 10.5194/essd-8-605-2016

[3] Caldeira K, Wickett ME. Oceanography: Anthropogenic carbon and ocean pH. Nature. 2003;425: 365–365. doi: 10.1038/425365a 14508477 10.1038/425365a

[4] Doney SC, Fabry VJ, Feely RA, Kleypas JA. Ocean acidification: The other CO2 problem. Annu Rev Marine Sci. 2009;1: 169–192. doi: 10.1146/annurev.marine.010908.163834 21141034 10.1146/annurev.marine.010908.163834

[5] Kroeker KJ, Kordas RL, Crim RN, Singh GG. Meta-analysis reveals negative yet variable effects of ocean acidification on marine organisms. Ecol Lett. 2010;13: 1419–1434. doi: 10.1111/j.1461-0248.2010.01518.x 20958904 10.1111/j.1461-0248.2010.01518.x

[6] Fabry VJ, Seibel BA, Feely RA, Orr JC. Impacts of ocean acidification on marine fauna and ecosystem processes. ICES J Mar Sci. 2008;65: 414–432. doi: 10.1093/icesjms/fsn048

[7] Kroeker KJ, Micheli F, Gambi MC. Ocean acidification causes ecosystem shifts via altered competitive interactions. Nat Clim Chang. 2012;3: 156–159. doi: 10.1038/nclimate1680

[8] Crawfurd KJ, Alvarez-Fernandez S, Mojica KDA, Riebesell U, Brussaard CPD. Alterations in microbial community composition with increasing *f*CO2: A mesocosm study in the eastern Baltic Sea. Biogeosciences. 2017;14: 3831–3849. doi: 10.5194/bg-14-3831-2017

[9] Schulz KG, Bach LT, Bellerby RGJ, Bermúdez Monsalve JR, Büdenbender J, Boxhammer T, et al Phytoplankton blooms at increasing levels of atmospheric carbon dioxide: Experimental evidence for negative effects on prymnesiophytes and positive on small picoeukaryotes. Front Mar Sci. 2017;4: 1–18. doi: 10.3389/fmars.2017.00064

[10] Lischka S, Büdenbender J, Boxhammer T, Riebesell U. Impact of ocean acidification and elevated temperatures on early juveniles of the polar shelled pteropod *Limacina helicina*: Mortality, shell degradation, and shell growth. Biogeosciences. 2011;8: 919–932. doi: 10.5194/bg-8-919-2011

[11] Riebesell U, Bach LT, Bellerby RGJ, Bermúdez Monsalve JR, Boxhammer T, Czerny J, et al Competitive fitness of a predominant pelagic calcifier impaired by ocean acidification. Nat Geosci. 2017;10: 19–23. doi: 10.1038/NGEO2854

[12] Sala MM, Aparicio FL, Balagué V, Boras JA, Borrull E, Cardelús C, et al Contrasting effects of ocean acidification on the microbial food web under different trophic conditions. ICES J Mar Sci. 2015;73: 670–679. doi: 10.1093/icesjms/fsv130

[13] Riebesell U, Schulz KG, Bellerby RGJ, Botros M, Fritsche P, Meyerhöfer M, et al Enhanced biological carbon consumption in a high CO_2_ ocean. Nature. 2007;450: 545–548. doi: 10.1038/nature06267 17994008 10.1038/nature06267

[14] Bellerby RGJ, Schulz KG, Riebesell U, Neill C, Nondal G, Heegaard E, et al Marine ecosystem community carbon and nutrient uptake stoichiometry under varying ocean acidification during the PeECE III experiment. Biogeosciences. 2008;5: 1517–1527. doi: 10.5194/bg-5-1517-2008

[15] Finkel ZV, Beardall J, Flynn KJ, Quigg A, Rees TAV, Raven JA. Phytoplankton in a changing world: Cell size and elemental stoichiometry. J Plankton Res. 2010;32: 119–137. doi: 10.1093/plankt/fbp098

[16] Kim J-M, Lee K, Shin K, Yang EJ, Engel A, Karl DM, et al Shifts in biogenic carbon flow from particulate to dissolved forms under high carbon dioxide and warm ocean conditions. Geophys Res Lett. 2011;38: L08612 doi: 10.1029/2011GL047346

[17] Engel A, Borchard C, Piontek J, Schulz KG, Riebesell U, Bellerby R. CO2 increases ^14^C primary production in an Arctic plankton community. Biogeosciences. 2013;10: 1291–1308. doi: 10.5194/bg-10-1291-2013

[18] Spilling K, Schulz KG, Paul AJ, Boxhammer T, Achterberg EP, Hornick T, et al Effects of ocean acidification on pelagic carbon fluxes in a mesocosm experiment. Biogeosciences. 2016;13: 6081–6093. doi: 10.5194/bg-13-6081-2016

[19] Engel A, Piontek J, Grossart HP, Riebesell U, Schulz KG, Sperling M. Impact of CO2 enrichment on organic matter dynamics during nutrient induced coastal phytoplankton blooms. J Plankton Res. 2014;36: 641–657. doi: 10.1093/plankt/fbt125

[20] Endres S, Galgani L, Riebesell U, Schulz KG, Engel A. Stimulated bacterial growth under elevated *p*CO2: Results from an off-shore mesocosm study. Dupont S, editor. PLoS ONE. 2014;9: e99228–8. doi: 10.1371/journal.pone.0099228 24941307 10.1371/journal.pone.0099228 PMC4062391

[21] Passow U. Transparent exopolymer particles (TEP) in aquatic environments. Prog Oceanogr. 2002;55: 287–333. doi: 10.1016/S0079-6611(02)00138-6

[22] Thornton DCO. Dissolved organic matter (DOM) release by phytoplankton in the contemporary and future ocean. Eur J Phycol. 2014;49: 20–46. doi: 10.1080/09670262.2013.875596

[23] Rossoll D, Bermúdez Monsalve JR, Hauss H, Schulz KG, Riebesell U, Sommer U, et al Ocean acidification-induced food quality deterioration constrains trophic transfer. Thrush S, editor. PLoS ONE. 2012;7: e34737–6. doi: 10.1371/journal.pone.0034737 22509351 10.1371/journal.pone.0034737 PMC3324536

[24] Schoo KL, Malzahn AM, Krause E, Boersma M. Increased carbon dioxide availability alters phytoplankton stoichiometry and affects carbon cycling and growth of a marine planktonic herbivore. Mar Biol. 2012;160: 2145–2155. doi: 10.1007/s00227-012-2121-4

[25] Sanders R, Henson SA, Koski M, La Rocha De CL, Painter SC, Poulton AJ, et al The biological carbon pump in the North Atlantic. Prog Oceanogr. 2014;129: 200–218. doi: 10.1016/j.pocean.2014.05.005

[26] Bach LT, Taucher J, Boxhammer T, Ludwig A, Achterberg EP, Algueró-Muñiz M, et al Influence of ocean acidification on a natural winter-to-summer plankton succession: First insights from a long-term mesocosm study draw attention to periods of low nutrient concentrations. Anil AC, editor. PLoS ONE. 2016;11: e0159068 EP–. doi: 10.1371/journal.pone.0159068 27525979 10.1371/journal.pone.0159068 PMC4985126

[27] Riebesell U, Czerny J, Bröckel von K, Boxhammer T, Büdenbender J, Deckelnick M, et al Technical Note: A mobile sea-going mesocosm system–new opportunities for ocean change research. Biogeosciences. 2013;10: 1835–1847. doi: 10.5194/bg-10-1835-2013

[28] Czerny J, Schulz KG, Krug SA, Ludwig A, Riebesell U. Technical Note: The determination of enclosed water volume in large flexible-wall mesocosms “KOSMOS.” Biogeosciences. 2013;10: 1937–1941. doi: 10.5194/bg-10-1937-2013

[29] Boxhammer T, Bach LT, Czerny J, Riebesell U. Technical note: Sampling and processing of mesocosm sediment trap material for quantitative biogeochemical analysis. Biogeosciences. 2016;13: 2849–2858. doi: 10.5194/bg-13-2849-2016

[30] Zark M, Riebesell U, Dittmar T. Effects of ocean acidification on marine dissolved organic matter are not detectable over the succession of phytoplankton blooms. Sci Adv. 2015;1: e1500531–e1500531. doi: 10.1126/sciadv.1500531 26601292 10.1126/sciadv.1500531 PMC4646806

[31] Kattner G. Storage of dissolved inorganic nutrients in seawater: Poisoning with mercuric chloride. Mar Chem. 1999;67: 61–66. doi: 10.1016/S0304-4203(99)00049-3

[32] Algueró-Muñiz M, Alvarez-Fernandez S, Thor P, Bach LT, Esposito M, Horn HG, et al Ocean acidification effects on mesozooplankton community development: Results from a long-term mesocosm experiment. PLoS ONE. 2017;12: e0175851 doi: 10.1371/journal.pone.0175851 28410436 10.1371/journal.pone.0175851 PMC5391960

[33] Johnson KM, Sieburth JM, Williams PJL, Brändström L. Coulometric total carbon dioxide analysis for marine studies: Automation and calibration. Mar Chem. 1987;21: 117–133. doi: 10.1016/0304-4203(87)90033-8

[34] Clayton TD, Byrne RH. Spectrophotometric seawater pH measurements: total hydrogen ion concentration scale calibration of m-cresol purple and at-sea results. Deep-Sea Res PT I. 1993;40: 2115–2129. doi: 10.1016/0967-0637(93)90048-8

[35] van Heuven S, Pierrot D, Rae JWB, Lewis E, Wallace DWR. CO2SYS v 1.1. MATLAB program developed for CO2 system calculations. ORNL/CDIAC-105b. Oak Ridge National Laboratory; 2011.

[36] Lueker TJ, Dickson AG, Keeling CD. Ocean *p*CO2 calculated from dissolved inorganic carbon, alkalinity, and equations for K1 and K2: validation based on laboratory measurements of CO2 in gas and seawater at equilibrium. Mar Chem. 2000;70: 105–119. doi: 10.1016/S0304-4203(00)00022-0

[37] Patey MD, Rijkenberg MJA, Statham PJ, Stinchcombe MC, Achterberg EP, Mowlem M. Determination of nitrate and phosphate in seawater at nanomolar concentrations. Trac-Trend Anal Chem. 2008;27: 169–182. doi: 10.1016/j.trac.2007.12.006

[38] Holmes RM, Aminot A, Kérouel R, Hooker BA, Peterson BJ. A simple and precise method for measuring ammonium in marine and freshwater ecosystems. Can J Fish Aquat Sci. 1999;56: 1801–1808. doi: 10.1139/f99-128

[39] Sharp JH. Improved analysis for “particulate” organic carbon and nitrogen from seawater. Limnol Oceangr. 1974;19: 984–989. doi: 10.4319/lo.1974.19.6.0984

[40] Hansen HP, Koroleff F. Determination of nutrients Methods of seawater analysis. Weinheim, Germany: Wiley-VCH Verlag GmbH; 1999 pp. 159–228. doi: 10.1002/9783527613984.ch10

[41] Gorsky G, Ohman MD, Picheral M, Gasparini S, Stemmann L, Romagnan J-B, et al Digital zooplankton image analysis using the ZooScan integrated system. J Plankton Res. 2010;32: 285–303. doi: 10.1093/plankt/fbp124

[42] Lehette P, Hernández-León S. Zooplankton biomass estimation from digitized images: A comparison between subtropical and Antarctic organisms. Limnol Oceanogr Methods. 2009;7: 304–308. doi: 10.4319/lom.2009.7.304

[43] Kiørboe T. Zooplankton body composition. Limnol Oceangr. 2013;58: 1843–1850. doi: 10.4319/lo.2013.58.5.1843

[44] Gismervik I. Stoichiometry of some marine planktonic crustaceans. J Plankton Res. 1997;19: 279–285. doi: 10.1093/plankt/19.2.279

[45] Hernández-León S, Montero I. Zooplankton biomass estimated from digitalized images in Antarctic waters: A calibration exercise. J Geophys Res. 2006;111: 307–6. doi: 10.1029/2005JC002887

[46] Garijo JC, Hernández-León S. The use of an image-based approach for the assessment of zooplankton physiological rates: A comparison with enzymatic methods. J Plankton Res. 2015;37: 923–938. doi: 10.1093/plankt/fbv056

[47] Biard T, Stemmann L, Picheral M, Mayot N, Vandromme P, Hauss H, et al In situ imaging reveals the biomass of giant protists in the global ocean. Nature. 2016;532: 504–507. doi: 10.1038/nature17652 27096373 10.1038/nature17652

[48] Hansell DA, Carlson CA. Net community production of dissolved organic carbon. Global Biogeochem Cycles. 1998;12: 443–453. doi: 10.1029/98GB01928

[49] Silyakova A, Bellerby RGJ, Schulz KG, Czerny J, Tanaka T, Nondal G, et al Pelagic community production and carbon-nutrient stoichiometry under variable ocean acidification in an Arctic fjord. Biogeosciences. 2013;10: 4847–4859. doi: 10.5194/bg-10-4847-2013

[50] R Core Team 2015. R: A language and environment for statistical computing [Internet]. Vienna, Austria. Available: http://www.R-project.org/

[51] Anderson MJ. A new method for non‐parametric multivariate analysis of variance. Austral Ecology. 2001;26: 32–46. doi: 10.1111/j.1442-9993.2001.01070.pp.x

[52] Anderson MJ, Walsh DCI. PERMANOVA, ANOSIM, and the Mantel test in the face of heterogeneous dispersions: What null hypothesis are you testing? Ecol Monogr. 2013;83: 557–574. doi: 10.1890/12-2010.1

[53] Bach LT, Alvarez-Fernandez S, Hornick T, Stuhr A, Riebesell U. Simulated ocean acidification reveals winners and losers in coastal phytoplankton. Dam HG, editor. PLoS ONE. 2017;12: e0188198–22. doi: 10.1371/journal.pone.0188198 29190760 10.1371/journal.pone.0188198 PMC5708705

[54] Amo YD, Brzezinski MA. The chemical form of dissolved Si taken up by marine diatoms. J Phycol. 1999;35: 1162–1170. doi: 10.1046/j.1529-8817.1999.3561162.x

[55] Thamatrakoln K, Hildebrand M. Silicon uptake in diatoms revisited: A model for saturable and nonsaturable uptake kinetics and the role of silicon transporters. Plant Physiol. 2008;146: 1397–1407. doi: 10.1104/pp.107.107094 18162598 10.1104/pp.107.107094 PMC2259041

[56] DeMaster D, Dunbar R, Gordon L, Leventer A, Morrison J, Nelson D, et al Cycling and Accumulation of Biogenic Silica and Organic Matter in High-Latitude Environments: The Ross Sea. Oceanography. 1992;5: 146–153. doi: 10.5670/oceanog.1992.03

[57] Nelson DM, DeMaster DJ, Dunbar RB, Smith WO. Cycling of organic carbon and biogenic silica in the Southern Ocean: Estimates of water‐column and sedimentary fluxes on the Ross Sea continental shelf. J Geophys Res. 1996;101: 18519–18532. doi: 10.1029/96JC01573

[58] Horn HG, Sander N, Stuhr A, Algueró-Muñiz M, Bach LT, Löder MGJ, et al Low CO2 sensitivity of microzooplankton communities in the Gullmar Fjord, Skagerrak: Evidence from a long-term mesocosm study. Doi H, editor. PLoS ONE. 2016;11: e0165800 doi: 10.1371/journal.pone.0165800 27893740 10.1371/journal.pone.0165800 PMC5125589

[59] Benitez-Nelson CR, Buesseler KO. Variability of inorganic and organic phosphorus turnover rates in the coastal ocean. Nature. 1999;398: 502–505. doi: 10.1038/19061

[60] Bronk DA, See JH, Bradley P, Killberg L. DON as a source of bioavailable nitrogen for phytoplankton. Biogeosciences. 2007;4: 283–296. doi: 10.5194/bg-4-283-2007

[61] White AE, Watkins-Brandt KS, Engle MA, Burkhardt B, Paytan A. Characterization of the rate and temperature sensitivities of bacterial remineralization of dissolved organic phosphorus compounds by natural populations. Front Microbiol. 2012;3: 276–13. doi: 10.3389/fmicb.2012.00276 22908008 10.3389/fmicb.2012.00276 PMC3415674

[62] Chen CC, Petersen JE, Kemp WM. Spatial and temporal scaling of periphyton growth on walls of estuarine mesocosms. Mar Ecol Prog Ser. 1997;155: 1–15. doi: 10.3354/meps155001

[63] Riebesell U, Lee K, Nejstgaard JC. Pelagic mesocosms In: Riebesell U, Fabry VJ, Hansson L, Gattuso J-P, editors. Guide to best practices in ocean acidification research and data reporting. Luxembourg; 2010 pp. 95–112. doi: 10.2777/58454

[64] Czerny J, Schulz KG, Boxhammer T, Bellerby RGJ, Büdenbender J, Engel A, et al Implications of elevated CO2 on pelagic carbon fluxes in an Arctic mesocosm study–an elemental mass balance approach. Biogeosciences. 2013;10: 3109–3125. doi: 10.5194/bg-10-3109-2013

[65] Martin-Jézéquel V, Hildebrand M, Brzezinski MA. Silicon metabolism in diatoms: Implications for growth. J Phycol. 2000;36: 821–840. doi: 10.1046/j.1529-8817.2000.00019.x

[66] Paul AJ, Bach LT, Schulz KG, Boxhammer T, Czerny J, Achterberg EP, et al Effect of elevated CO2 on organic matter pools and fluxes in a summer Baltic Sea plankton community. Biogeosciences. 2015;12: 6181–6203. doi: 10.5194/bg-12-6181-2015

[67] Czerny J, Schulz KG, Ludwig A, Riebesell U. Technical Note: A simple method for air–sea gas exchange measurements in mesocosms and its application in carbon budgeting. Biogeosciences. 2013;10: 1379–1390. doi: 10.5194/bg-10-1379-2013

[68] Strong AL, Lowry KE, Brown ZW, Mills MM, van Dijken GL, Pickart RS, et al Mass balance estimates of carbon export in different water masses of the Chukchi Sea shelf. Deep Sea Res Part 2 Top Stud Oceanogr. 2016;130 IS -: 88–99. doi: 10.1016/j.dsr2.2016.05.003

[69] Howarth RW, Marino R, Lane J, Cole JJ. Nitrogen fixation in freshwater, estuarine, and marine ecosystems. 1. Rates and importance. Limnol Oceangr. 1988;33: 669–687. doi: 10.4319/lo.1988.33.4part2.0669

[70] Stal LJ, Albertano P, Bergman B, Bröckel von K, Gallon JR, Hayes PK, et al BASIC: Baltic Sea cyanobacteria. An investigation of the structure and dynamics of water blooms of cyanobacteria in the Baltic Sea—responses to a changing environment. Cont Shelf Res. 2003;23: 1695–1714. doi: 10.1016/j.csr.2003.06.001

[71] Ohlendieck U, Gundersen K, Meyerhöfer M, Fritsche P, Nachtigall K, Bergmann B. The significance of nitrogen fixation to new production during early summer in the Baltic Sea. Biogeosciences. 2007;4: 63–73. doi: 10.5194/bg-4-63-2007

[72] Eberlein T, Wohlrab S, Rost B, John U, Bach LT, Riebesell U, et al Effects of ocean acidification on primary production in a coastal North Sea phytoplankton community. Vopel KC, editor. PLoS ONE. 2017;12: e0172594–15. doi: 10.1371/journal.pone.0172594 28273107 10.1371/journal.pone.0172594 PMC5342202

[73] Sambrotto RN, Savidge G, Robinson C, Boyd P, Takahashi T, Karl DM, et al Elevated consumption of carbon relative to nitrogen in the surface ocean. Nature. 1993;363: 248–250. doi: 10.1038/363248a0

[74] Toggweiler JR. Carbon overconsumption. Nature. 1993;363: 210–211. doi: 10.1038/363210a0

[75] Taucher J, Haunost M, Boxhammer T, Bach LT, Alguer Mu iz MA, Riebesell U. Influence of ocean acidification on plankton community structure during a winter-to-summer succession: An imaging approach indicates that copepods can benefit from elevated CO2 via indirect food web effects. Ianora A, editor. PLoS ONE. 2017;12: e0169737–23. doi: 10.1371/journal.pone.0169737 28178268 10.1371/journal.pone.0169737 PMC5298333

[76] Thingstad TF, Krom MD, Mantoura RFC, Flaten GAF, Groom S, Herut B, et al Nature of phosphorus limitation in the ultraoligotrophic eastern Mediterranean. Science. 2005;309: 1068–1071. doi: 10.1126/science.1112632 16099984 10.1126/science.1112632

[77] Sswat M, Stiasny MH, Taucher J, Algueró-Muñiz M, Bach LT, Jutfelt F, et al Food web changes under ocean acidification promote herring larvae survival. Nat Ecol Evol. 2018;10: 1–8. doi: 10.1038/s41559-018-0514-6 29556079 10.1038/s41559-018-0514-6

