# Supplementary material for: Enhanced transfer of organic matter to higher trophic levels caused by ocean acidification and its implications for export production: A mass balance approach
Source: PLoS One. 2018 May 25;13(5):e0197502. doi: 10.1371/journal.pone.0197502 (PMC5969766; doi:10.1371/journal.pone.0197502)
Supplement: S1 Fig — Solid lines show mean values of the biogenic silica (BSi) to particulate carbon (TPC) ratio in (A) the water column and (B) sediment trap samples of the ambient (blue) and high (red) CO2 treatment. Coloured areas indicate standard deviation of the replicated (n = 5) treatments. Roman numbers denote the different phases of the experiment. (PDF) [file pone.0197502.s001.pdf]

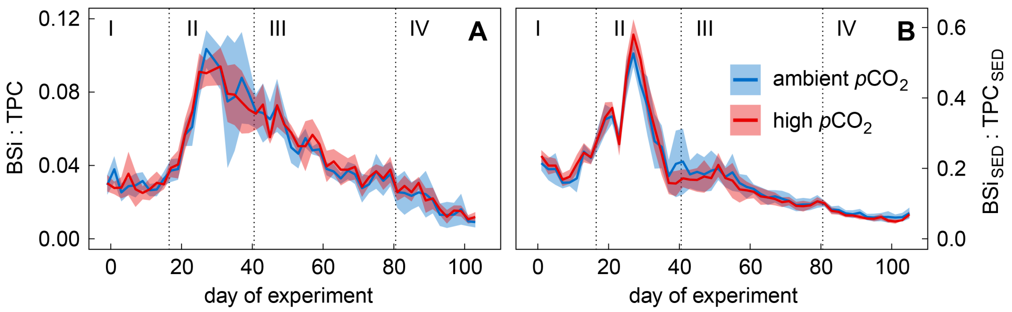

S1 Fig. Time course of the biogenic silica to total particulate carbon ratio.

Solid lines show mean values of the biogenic silica (BSi) to particulate carbon (TPC) ratio in (A) the water column and (B) sediment trap samples of the ambient (blue) and high (red) CO_2 treatment. Coloured areas indicate standard deviation of the replicated (n = 5) treatments. Roman numbers denote the different phases of the experiment.
