# Supplementary material for: Enhanced transfer of organic matter to higher trophic levels caused by ocean acidification and its implications for export production: A mass balance approach
Source: PLoS One. 2018 May 25;13(5):e0197502. doi: 10.1371/journal.pone.0197502 (PMC5969766; doi:10.1371/journal.pone.0197502)
Supplement: S2 Fig — Solid lines, dotted lines, and dashed lines represent the three size classes of total biogenic silica (BSi), the fraction >200 μm, and the fraction <200 μm respectively. All lines represent mean values of the (A) ambient and (B) high CO2 treatment. Roman numbers denote the different phases of the experiment. (PDF) [file pone.0197502.s002.pdf]

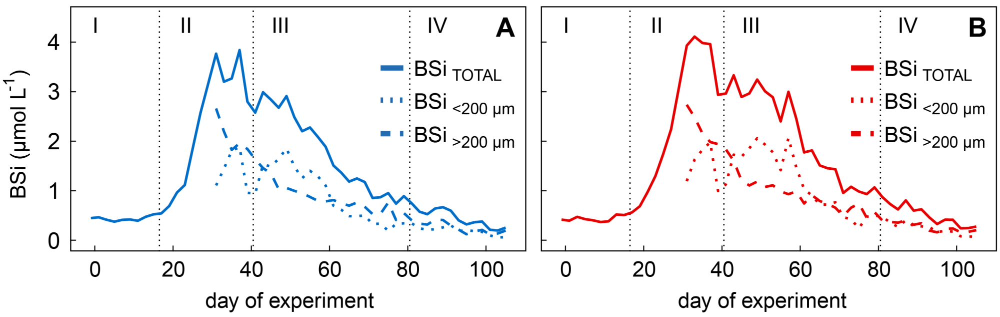

S2 Fig. Time course of different size classes of biogenic silica.

Solid lines, dotted lines, and dashed lines represent the three size classes of total biogenic silica (BSi), the fraction > 200 µm, and the fraction < 200 µm respectively. All lines represent mean values of the (A) ambient and (B) high CO_2 treatment. Roman numbers denote the different phases of the experiment.
