# Supplementary material for: Enhanced transfer of organic matter to higher trophic levels caused by ocean acidification and its implications for export production: A mass balance approach
Source: PLoS One. 2018 May 25;13(5):e0197502. doi: 10.1371/journal.pone.0197502 (PMC5969766; doi:10.1371/journal.pone.0197502)
Supplement: S3 Fig — Dashed lines show net changes of dissolved organic carbon (DOC, yellow) and net community production of carbon (NCP, blue/red) as average values of (A) ambient and (B) high CO2 mesocosms. Solid lines of the same colour code show strongly smoothed data (moving average of nine), with an adjusted reference period for calculation of net changes to t1 –t17. Accordingly, smoothed data sets do not start before day 9. Roman numbers denote the different phases of the experiment. (PDF) [file pone.0197502.s003.pdf]

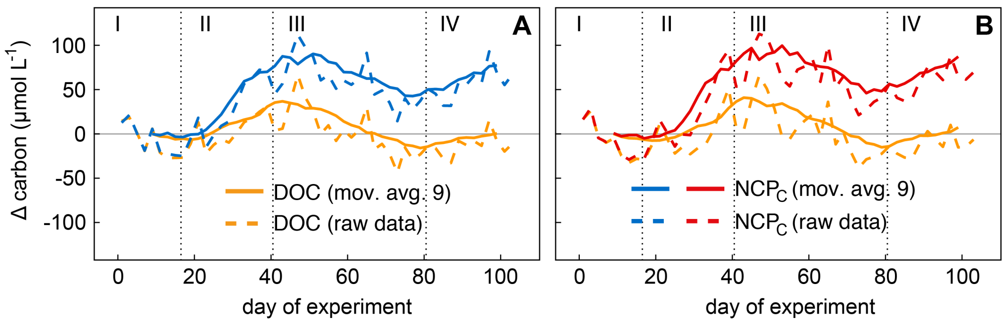

S3 Fig. Moving average of dissolved organic carbon and net community production.

Dashed lines show net changes of dissolved organic carbon (DOC, yellow) and net community production of carbon (NCP, blue/red) as average values of (A) ambient and (B) high CO_2 mesocosms. Solid lines of the same colour code show strongly smoothed data (moving average of nine), with an adjusted reference period for calculation of net changes to $t_1 - t_{17}$. Accordingly, smoothed data sets do not start before day 9. Roman numbers denote the different phases of the experiment.
