# Supplementary material for: Enhanced transfer of organic matter to higher trophic levels caused by ocean acidification and its implications for export production: A mass balance approach
Source: PLoS One. 2018 May 25;13(5):e0197502. doi: 10.1371/journal.pone.0197502 (PMC5969766; doi:10.1371/journal.pone.0197502)
Supplement: S4 Fig — Section of a high-resolution image, taken from a sediment trap subsample of Mesocosm 4 (high CO2) on t65 (Phase III). The highly abundant round objects are cells of the large diatom Coscinodiscus concinnus (Smith, 1856). (PDF) [file pone.0197502.s004.pdf]

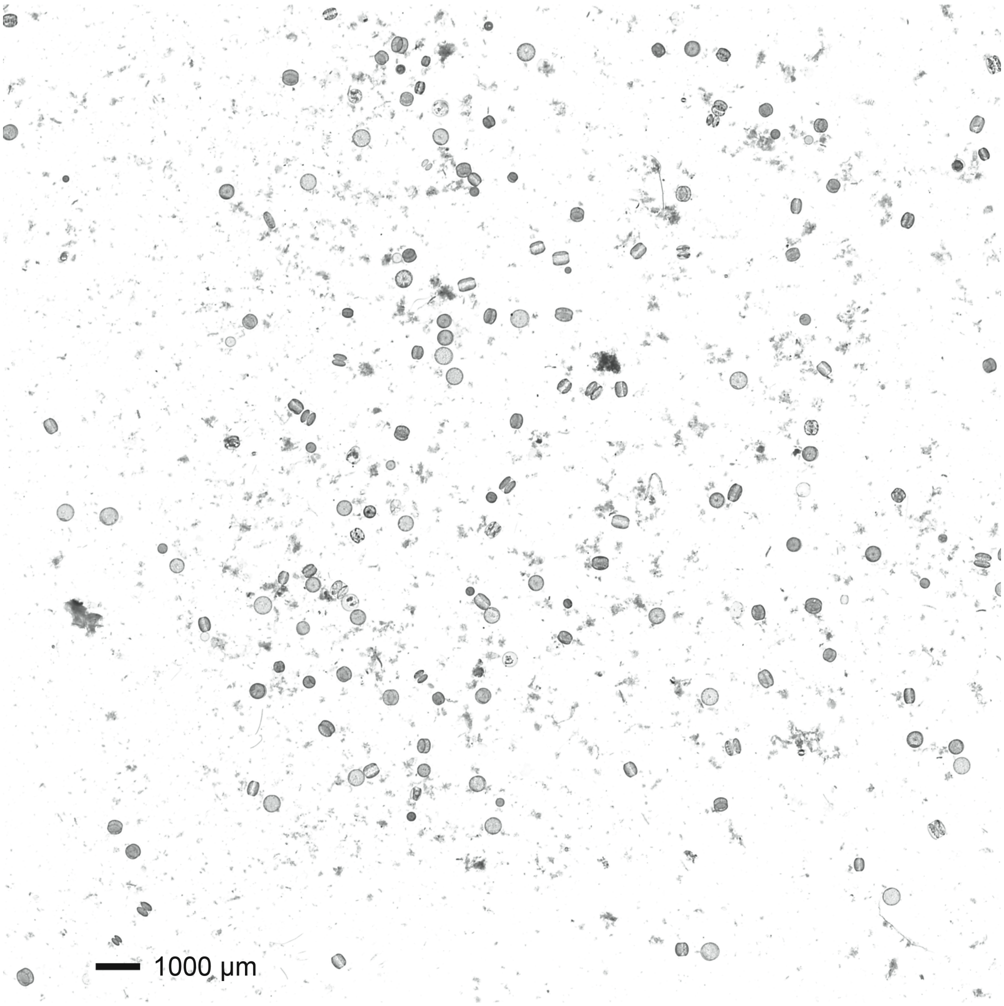

S4 Fig. High-resolution image of a typical sediment trap sample during Phase III.

Section of a high-resolution image, taken from a sediment trap subsample of Mesocosm 4 (high CO_2) on t_{65} (Phase III). The highly abundant round objects are cells of the large diatom *Coscinodiscus concinnus*.
